# Essential role of p21^Waf1/Cip1^ in the modulation of post-traumatic hippocampal Neural Stem Cells response

**DOI:** 10.1186/s13287-024-03787-0

**Published:** 2024-07-06

**Authors:** Francesco Chiani, Valentina Mastrorilli, Nicole Marchetti, Andrea Macioce, Chiara Nappi, Georgios Strimpakos, Miriam Pasquini, Alessia Gambadoro, Jonathan Isacco Battistini, Debora Cutuli, Laura Petrosini, Sara Marinelli, Raffaella Scardigli, Stefano Farioli Vecchioli

**Affiliations:** 1 Institute of Biochemistry and Cell Biology, IBBC, CNR, Monterotondo, Rome, Italy; 2 PhD Course in Sciences of Nutrition, Aging, Metabolism and Gender Pathologies, Catholic University of Roma, 00100 Rome, Italy; 3 Instituto de Neurosciencias, Universidad Miguel-Hernandez, Alicante, Spain; 4 Department of Psychology, Sapienza University of Rome, Via dei Marsi 78, 00185 Rome, Italy; 5 IRCCS Fondazione Santa Lucia, Via Ardeatina 306, 00179 Rome, Italy; 6 European Brain Research Institute (EBRI), Viale Regine Elena, 00161 Rome, Italy; 7 Institute of Translational Pharmacology, National Research Council, Rome, Italy

**Keywords:** p21, Adult Neural Stem Cells, Adult hippocampal neurogenesis, Traumatic brain injury, Neural regeneration, Working memory

## Abstract

**Background:**

Traumatic Brain Injury (TBI) represents one of the main causes of brain damage in young people and the elderly population with a very high rate of psycho-physical disability and death. TBI is characterized by extensive cell death, tissue damage and neuro-inflammation with a symptomatology that varies depending on the severity of the trauma from memory loss to a state of irreversible coma and death. Recently, preclinical studies on mouse models have demonstrated that the post-traumatic adult Neural Stem/Progenitor cells response could represent an excellent model to shed light on the neuro-reparative role of adult neurogenesis following damage. The cyclin-dependent kinase inhibitor p21^Waf1/Cip1^ plays a pivotal role in modulating the quiescence/activation balance of adult Neural Stem Cells (aNSCs) and in restraining the proliferation progression of progenitor cells. Based on these considerations, the aim of this work is to evaluate how the conditional ablation of p21^Waf1/Cip1^ in the aNSCS can alter the adult hippocampal neurogenesis in physiological and post-traumatic conditions.

**Methods:**

We designed a novel conditional p21^Waf1/Cip1^ knock-out mouse model, in which the deletion of p21^Waf1/Cip1^ (referred as p21) is temporally controlled and occurs in Nestin-positive aNSCs, following administration of Tamoxifen. This mouse model (referred as p21 cKO mice) was subjected to Controlled Cortical Impact to analyze how the deletion of p21 could influence the post-traumatic neurogenic response within the hippocampal niche.

**Results:**

The data demonstrates that the conditional deletion of p21 in the aNSCs induces a strong increase in activation of aNSCs as well as proliferation and differentiation of neural progenitors in the adult dentate gyrus of the hippocampus, resulting in an enhancement of neurogenesis and the hippocampal-dependent working memory. However, following traumatic brain injury, the increased neurogenic response of aNSCs in p21 cKO mice leads to a fast depletion of the aNSCs pool, followed by declined neurogenesis and impaired hippocampal functionality.

**Conclusions:**

These data demonstrate for the first time a fundamental role of p21 in modulating the post-traumatic hippocampal neurogenic response, by the regulation of the proliferative and differentiative steps of aNSCs/progenitor populations after brain damage.

**Supplementary Information:**

The online version contains supplementary material available at 10.1186/s13287-024-03787-0.

## Introduction

TBI represents one of the leading causes of death worldwide, as well as being a serious social, economic and health problem globally. It is the main cause of coma, plays a key role in disabilities due to traumatic events and is the most frequent cause of brain damage in children and young adults [1, 2]. In Europe, head trauma is responsible for more years of disability than any other cause [3] while in the United States approximately 80 thousand people every year are victims of brain damage [4, 5]. Following TBI, primary brain lesions are observed when tissues and blood vessels are stretched and compressed [6, 7]; these primary injuries are followed by secondary damages, a complex set of cellular processes and biochemical cascades that occur from a few minutes to several days following the trauma and can cause a drastic worsening of the patient's general condition with a high probability of death in more cases serious [8, 9]. The hippocampus is one of the brain region most vulnerable to cell death and synaptic dysfunction after TBI, with consequent impairment in learning and memory processes [10, 11]. In this regard, the post-traumatic increase of proliferation of neural stem/progenitor cells observed in the hippocampal dentate gyrus could represent a pivotal compensatory and regenerative mechanism after TBI-induced neuronal death. [12, 13]. However, the extent of the proliferative rate as well as the ability of newly generated neurons to mature, survive and integrate in a post-traumatic environment varies greatly depending on the severity of the TBI [13]. Likewise, the molecular mechanisms that regulate the different post-traumatic proliferative steps of NSCs/progenitor cells is still far to be fully elucidated.

The process of adult neurogenesis within the two main rodent neurogenic niches (subventricular zone and hippocampal dentate gyrus—DG) originates from aNSCs, which are committed cells giving rise neural and glial cells in the brain [14]. Within the DG, aNSCs are embedded in highly specialized niche that finely regulate their maintenance, activation and production of differentiated progeny [15–17]. Most of the aNSCs are in a state of quiescence, i.e. a stage of reversible cell cycle arrest, with the specific role of preserving the longevity of the pool [18, 19], providing a source of new cells during regenerative response after brain injury [20, 21]. Local signaling environment or internal (i.e. corticotropin-releasing hormone, [22]) and external (i.e. physical activity, [23]) stimuli, trigger quiescent aNSCs to enters in proliferation, giving rise neural precursors [14]. Once exited from cell cycle only a fraction of young differentiated neurons survives at two waves of apoptosis [24] and mature into new neurons functionally active in hippocampus-dependent learning and memory processes [25]. Over time proliferating aNSCs return to quiescence instead of differentiating [26] and quiescent stem cells progressively lose their ability to be recruited in the cell cycle. Moreover, a recent study has identified a subpopulation of NSCs undergoing asynchronous decline and showing early molecular aging [27]. All these events induce a gradual decrease in the aNSCs pool size with the consequent disappearance of neurogenic activity in the adult and aged brain [26, 27]. Hence, the study of endogenous molecular regulators and exogenous environmental factors capable of modulating the quiescence/recruitment transition of NSCs represents a challenge in the understanding of the long-term behaviour of NSCs and in the attempt to slow down, or counteract its exhaustion [28, 29]. In this regard, a pivotal mechanism underlying the fate of NSCs is represented by the cell cycle [30]. Indeed, it has been widely demonstrated that some of the main cell cycle regulators, the cyclin-dependent kinase inhibitors, p21, p27 and p57 are able to control the quiescence of NSCs, promoting cell cycle arrest by the inhibition of cyclin-dependent kinase activity [31, 32]. Within this family of cell cycle inhibitors, the most studied is the p21 protein, whose main role is to keep the NSCs in quiescence and to restrain progenitor proliferation [33]. In this regard, several studies have shown that the constitutive loss of p21 induces a rapid activation of quiescent NSCs at post-natal age, associated with reduction of NSC self-renewal capacity and with the appearance of replicative stress in the hyper-proliferating progenitors [34–37]. These events provoke an accelerated exhaustion of the stem cell pool, impaired neurogenesis and aged phenotype in the adult neurogenic niches [37, 38]. Remarkably, our recent studies have shown that an environmental factor such as physical activity is able to reactivate the recruitment and expansion of NSCs within the subventricular zone (SVZ) of p21 knockout mice inducing the increase of subventricular neurogenesis and the enhancement of the post-traumatic neurogenic response [37–39].

While there is an extensive literature describing the role of p21 in embryonic and postnatal murine neurogenic niches, there is no evidence of its role in adulthood and even less in a post-traumatic condition. These considerations prompted us to verify whether the p21 modulation in aNSCs of DG might represent a useful tool for enhancing adult neurogenesis in physiological conditions and also during the neuro-regenerative response to the brain injury. To this aim, we generated a new p21 conditional knockout mouse by the insertion of two loxp sites flanking the exon 1 of the p21 gene, with the adoption of Crispr-Cas9 technology. To target the specific deletion of p21 in the NSCs, the p21 loxP mouse has been crossed with the Nestin Cre::ER^T2^ mice model. In these Cre-Lox mice model, the injection of Tamoxifen induced the deletion of the p21 gene selectively in the Nestin expressing aNSCs of the adult neurogenic niches.

Our results demonstrated that under physiological conditions the deletion of p21 in aNSCs of DG causes a long-term increase in adult neurogenesis leading to a hippocampus-dependent improvement of specific behavioural task. In vitro assay with neurospheres obtained from DG-derived aNSCs confirms the strong activation and proliferative capacity in aNSCs lacking p21. Conversely, after traumatic brain injury, p21 deletion in aNSCs induces hyper-activation and proliferation of NSCs followed by rapid depletion of aNSCs pool and a steep decline in hippocampal neurogenesis. These data demonstrate for the first time a fundamental role of p21 in modulating the post-traumatic neurogenic response by the regulation of the proliferative and differentiative steps of NSCs/progenitor populations in a post-traumatic context.

## Material and methods

For full description of experimental procedures please see the Additional file 1.

### ***Generation of p21***^***floxed***^*** mice***

#### Allele design

**Fig. 1:**
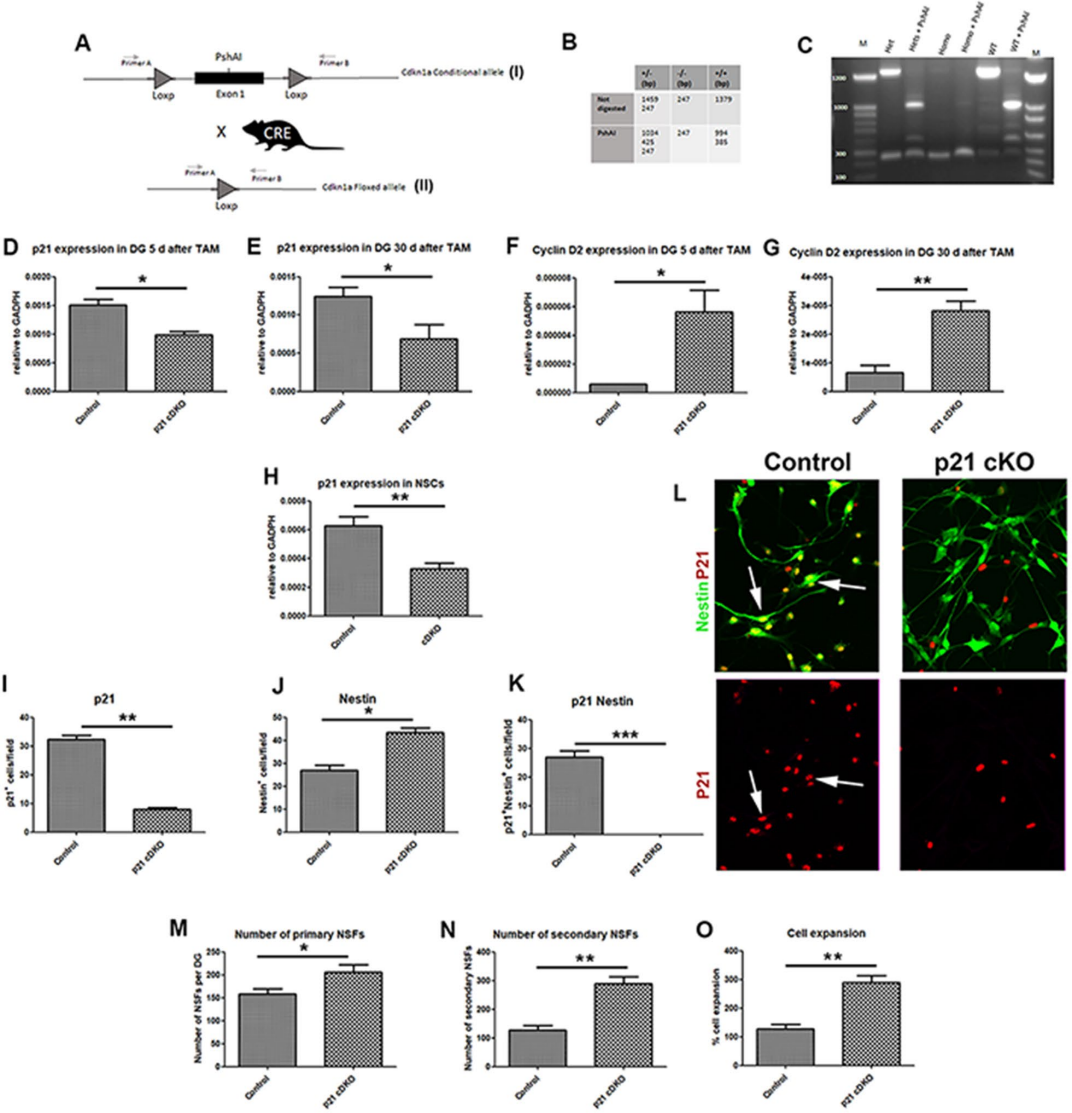
Generation and characterization of p21 cKO mice model. **A** Scheme of the putative synthetic allele before (I) and after (II) after Cdkn1a conditional mouse was bred with an ACT-CRE deleter. Loxp was inserted in Cis configuration. The breeding with a CRE deleter mouse lead to excision of the Exon1 of the Cdkn1a gene. **B** Sizes of the expected bands after Long Range PCR performed on: Heterozygotes mouse (Het), Homozygote mouse (Homo) and WT mouse (from the same litter). Expected additional bands were reported after PshAI restriction of the amplicons. **C** 1% Uncropped full-length agarose gel of the samples obtained by Long Range PCR using Primer A and B. As an additional control half of each sample was digested with PshAI restriction enzyme (PshAI). **D-E** rt-PCR analysis revealing the levels of p21 gene expression in the DG of p21 cKO and Control mice after 5 (**D**) and 28 (**E**) days after TAM injection. **F-G** Levels of expression of Cyclin D2 gene in the DG of p21 cKO and Control mice after 5 (**F**) and 28 (**G**) days after TAM injection. **H** Level of expression of p21 gene in the neurospheres obtained from p21 cKO and Control mice after 5 TAM injections. **I-J** Quantification of number of in vitro cultured NSCs isolated from Control and p21 cKO mice and immunostained for p21 (**I**), Nestin (**J**) and p21/Nestin (**K**) antibodies. **(L)** Representative images illustrating the decreased number of p21 (red), the increased number of Nestin (green), and the absence of p21 (red) in the Nestin^+^ cells (green) in the p21 cKO mice compared to the Control mice. **M** Number of clonal neurospheres derived from DG from the different experimental groups 5 days after TAM injection. Relative to the Control mice, neurospheres generated from p21 cKO mice largely increased. **N** Graph showing the number of secondary neurospheres (DIV 14), with a large enhancement of number neurospheres derived from p21 cKO mice. **O** Percentage of cell expansion of neurospheres expressed as the total number of cells at the end of the culture at the second passage divided by the initial number of cells. Compared with the Control conditions, a consistent expansion in the p21 cKO occurred. Statistical significance: *p < 0.05, **p < 0.01, ***p < 0.001, Mann Whitney Test. Confocal images magnification 20x. Expression analysis: N = 4 animals/groups. In vitro analysis: N = 5 animals/group

We identified exons 1 (ENSE00003747087) as critical exon of p21 gene, and we planned to insert two loxp sequences into the intron 1 and 2 far enough from the cis splicing sequences, in order to avoid splicing cis sequences perturbation (Fig. 1A). The detailed allele design is explained in Additional file 1  and Additional file 2: Fig. 1A, and described by Yang et al. [40].

#### Microinjection data

CrispR/Cas9 Technology was applied to obtain the desired allele following the Yang Protocol [40]. In brief, in vitro transcribed mRNA capped and polyadenylated Cas9 were microinjected together with pSgRNA and dSgRNA and ssDNA templates to into a total of 766 embryos, with a survival rate close to 25% (Additional file 2: Table 1). Zygotes were microinjected in the pronuclei and then were transferred to pseudo-pregnant CD1 females. The detailed informations on microinjection are displayed in Additional file 1.

#### Null allele induction by CRE deleter breeding

P21 floxed Allele cis design was also confirmed in F1 mice, by crossing with an ubiquitously expressing Act-CRE deleter mouse [41]. F1 was seletected GFP negative, but with the CRE recombinase event obtained [41].

### Experimental animals

Nes-cre/ERT2 mice** (**C57BL/6-Tg(Nes-cre/ERT2KEisc/J, Strain #016261) were obtain from Jackson Laboratory (RRID:IMSR_JAX:016261). The colony of p21 cKO mice (that is, Nes-CreERT2; p21^fl/fl^ mice) was maintained by interbreeding Nes-CreERT2; p21^fl/fl^ mice and p21^fl/fl^ mice. To induce CreERT2-mediated recombination of p21 in neural stem cells in the adult brain, mice (Nes-CreERT2; p21^fl/fl^) of at least 8 weeks of age were given 4 mg Tamoxifen (TAM, Sigma, T-5648) intra-peritoneally, once a day for 5 consecutive days. TAM solution was prepared in corn oil containing 10% ethanol. Tamoxifen solution was made fresh prior to the first injection of each animal and was stored at 4 °C for no more than one week. Control mice (Nes-CreERT2; p21^fl/fl^) were injected for 5 days with vehicle (corn oil containing 10% ethanol). Mice were housed four to five per cage in a 12 h (6 a.m. to 6 p.m.) light–dark colony room at 22 °C and had free access to food and water. Genomic extraction of DNA followed by genotyping was carried out from tail samples and performed as previously described [23, 37]. All animal experiments complied with the Italian ethical committee, which approve all experimental procedures (No: 785/19) and were carried out in accordance with EU Directive 2010/63/EU for animal experiments. This work has been carried out in line with the ARRIVE guidelines 2.0.

### Experimental procedures

A total of 220 male mice, ranging from 8 to 12 weeks of age were used for all the experimental procedures. The number of animals used in this study was determined in compliance with European Animal Laboratory regulations and was approved in the experimental protocol submitted to both the internal Animal Welfare Committee and the Italian Ministry of Health. Specifically, we conducted a priori sample size calculation (Power Analysis) based on a standard experiment (behavioral analysis). We considered four experimental groups (CTRL SHAM, CTRL TBI, p21 cKO SHAM, p21 cKO TBI) for a repeated measures ANOVA with three measurements (as in NOR) and ensured an adequate study power (1 − β) of 0.8 (80%). Power analysis is illustrated in Additional file 2: Fig. 2A; the total sample size is 40 (N = 10–12/group). For ex-vivo experiments, where individual variability is limited, in alignment with the 3R principle, we draw on our extensive experience in these methods and subject matter, employing the minimum number of animals necessary to obtain scientifically sound results.

Animals were given 5 injections of BrdU (95 mg/kg) the day after the last TAM/vehicle injection and subsequently sacrificed at 5, 28 and 100 days thereafter for immunofluorescence analyses. For animals not subjected to TBI, cognitive tests (Open Field, Elevated Plus Maze, Morris Water Maze and Novel Object Recognition) were performed 56 days post TAM. c-Fos analysis were performed 1 h after the probe trial of Morris Water Maze and 1 h after the NOR task. For animals subjected to TBI, the cognitive tests (Open Field, Elevated Plus Maze, and Novel Object Recognition) were carried out 30 days post TAM and TBI, while the motor test (Rotarod) was carried out 5, 15 and 30 days after TAM and TBI. We analyzed 5 animals for groups in the immunofluorescence studies and 8–12 animals/group for behavioural tests (The experimetal procedure is shown in Additional file 2: Fig. 2B).

*Traumatic brain injury* 3-month-old male animals were injected with TAM/vehicle and then BrdU as described above, subjected to an ipsilateral Controlled Cortical Injury (CCI) and sacrificed at 5, 15 and 30 days after CCI for immunofluorescence analyses. Mice were anesthetized with isoflurane and positioned within a mouse stereotaxic frame. Following a longitudinal skin incision, a 3 mm diameter craniotomy was performed at the following stereotaxic coordinates from Paxinos: antero-posterior: -2.3 mm; lateral 2 mm from Bregma [42]. Traumatic brain injury was performed at the cortical level with a flat, 3-mm diameter metal tip attached to the CCI device (PinPoint Precision Impactor, Stoelting, Wood Dale, IL, USA), at an impact speed of 3 m/sec, time of impact of 150 ms and a depth of 1 mm below the dura (Ctrl TBI and p21 cKO TBI, Fig. 7B; the experimental timing of TBI and the lesion impact are shown in Additional file 2: Fig. 2C, D). After the impact, the animals were sutured with absorbable suture thread, housed in their home cage and put on a heated plate for 3/4 h in order to control their body temperature during recovery from anesthesia. Animals subjected to the surgical procedures described above without cortical impact represented the SHAM groups (Ctrl SHAM and p21 cKO SHAM).

### Behavioural testing

#### Cognitive behaviour

All tests were performed between 10:00 a.m. and 06:00 p.m. All animals were subjected to handling habituation prior to the behavioral testing. Mice were tested in a pseudo-random order during each different task.

*Open Field* Spontaneous exploratory locomotor activity and thigmotaxis in the open field were used as a general measure of motor function and anxiety-related behaviors, respectively [43]. Locomotor activity was measured by distance travelled and average speed over the 20 min of exploration, where a large distance travelled and increased velocity are indicative of hyperactivity [44]. Additional file 1 shows the detailed Open Field Protocol.

*Elevated Plus Maze* The EPM was used to assess anxiety-like behavior. Each mouse was placed in the central arm crossing area (10 × 10 cm) and was given 5 min to freely explore on the EPM. The duration spent in the open and closed arms was automatically recorded by a video camera mounted on the ceiling facing the center of the maze. The complete precedure of EPM is present in Additional file 1.

*Morris Water Maze* The protocol consisted of a 16-trial Place phase and a 1-trial Probe phase. During the Place phase, mice from Control and p21 cKO groups were trained to locate a hidden platform using distal cues on four blocks of 90 s-trials each day for 4 days. During the 15–20 min inter-trial interval mice were put in their home cages. On the 5th day, spatial memory was then assessed during the Probe phase consisting of a trial performed 24 h after the final training trial. The complete procedure of MWM is displayed in  Additional file 1.

*Novel Object Recognition* This test is widely validated to measure recognition memory in rodents that exploits their natural tendency to explore novel items [45] and is strictly dependent on hippocampal integrity [46–48]. The apparatus consisted of a rectangular chamber made of transparent Plexiglas (56 × 42 × 21 cm). The test consisted of an habituation, a study phase and two test trials, one within 1-min of delay, the second one 3 h later. During habituation, mice were allowed to explore the empty chamber for 10 min. Afterwards, the mice were put in their home cages for 3 min. Then, during study phase, they were placed for 5 min in the chamber containing two identical objects (objects 1 and 2; two white Plexiglas 6.5-cm diameter spheres fixed to a 4 mm thick transparent squared base). One minute later, during the test trial, the mice were once again placed for 5 min to the chamber containing one familiar object (object 2) and a novel object (object 3: a white cube of 5.5-cm side fixed to a 4 mm thick transparent squared base) to test short-term recognition memory (test trial). Three hours later, mice were once again placed for 5 min in the chamber containing the familiar object (object 2) and the new object 4 (a white pyramidal prism fixed to a 4 mm thick transparent squared base). Contact time was considered to have occurred when the animal explored the object for at least 1 s. To balance eventual side bias, each animal was put in the testing chamber from the opposite long sides of the apparatus (with the snout against the wall), so that if for one animal the object 1 was on the right and the novel objects on the left, conversely for another animal inserted in the apparatus from the opposite side the object 1 was on the left and the novel objects on the right. The arena was cleaned with a solution of 70% ethanol after each trial to minimize olfactory signals. A video camera connected to a monitor and to the image analyzer (EthoVision XT, Noldus, The Netherlands) was placed on top of the apparatus. To assess novel object recognition (novelty) skills we calculated the percentage of the time spent exploring the new vs. familiar object during trials.

#### Motor behaviour

*Rotarod* The test was performed 5, 14 and 30 days after TBI. The latency to fall off the rotating rod was recorded. All animals were tested 3 times within an interval of at least 10 min between tests. Data were presented as the mean value from 3 tests. The Additional file 1 contains the full procedure of Rotarod.

### Immunohistochemistry

At the time point described in Experimental Procedures Section, the animals were sacrificed by trans-cardiac perfusion with 4% paraformaldehyde (PFA) in phosphate-buffered saline (PBS); the brains were collected and kept overnight at − 4 °C in PFA. They were subsequently equilibrated in sucrose dissolved at 30% in PBS 1X and finally cryopreserved at − 80 °C. Slicing of the brain area containing the hippocampal Dentate Gyrus was carried out by embedding the brain in Tissue-Tek OCT (Sakura, Torrence, CA, USA) and then cut in coronal section using a cryostat at − 25 °C throughout the whole rostro-caudal extent of the hippocampal Dentate Gyrus approximately (from Bregma − 1.34 to − 3.08, 42). The coronal sections were processed in a 1-in-6 series protocol at a 40 µm thickness then stained for multiple fluorescence labelling [49, 50]. The detailed informations relative to the immunocytochemical procedures are present in Additional file 1.

### Cell counting

Slices were collected using systematic random sampling. The brain was coronally sliced in rostro-caudal direction. Approximately total of 60 coronal sections with 40 μm of thickness were obtained from each brain; about 1-in-6 series of sections (each slice thus spaced 240 μm apart from the next) were analyzed by confocal microscopy and used to count the number of cells expressing the different markers throughout the rostro-caudal extent of the dorsal hippocampus. The estimation of the total DG cell numbers was obtained by multiplying the average number of positive cells per section for 240 which represents the distance between the section analyzed. For the details of cell counting see Additional file 1.

### In vitro analysis

With the aim of evaluating how the conditional deletion of p21 can modify the properties of self-renewing, proliferation and aNSCs pool expansion, 2-month-old p21 cKO mice injected for 5 days with TAM/vehicle were euthanized by CO_2_ 5 days after the last injection of TAM/vehicle and the brains were successively removed. DGs were dissected out and cells were isolated. The isolation of bilateral DG was performed under a stereomicroscope, following a described procedure [51]. To perform a neurosphere assay, cells isolated from DG were cultured under clonal conditions, in which neurospheres are generated from single cells and serving as an index of the number of in vivo neural stem cells. The complete procedure of in vitro analysis is present in Additional file 1.

#### Expression analysis by rtPCR

Total RNA from DG and secondary neurospheres was obtained by homogenizing the tissues with a tissue homogenizer (OMNI GLH INTERNATIONAL; power 1; 5 s) in TriReagent (Sigma-Aldrich Cat#: T9424). The RNA extraction was performed following TriReagent manufacturer’s protocol. We analyzed the expression of p21, Cyclin D1 and Cyclin D2. The sequences of murine expression oligonucleotides for RT-qPCR are listed in Additional file 2: Table 3. All Ct values were obtained in duplicate or triplicate and the analysis of output values was made using standard ΔΔCt method. The details of expression analysis are shown in Additional file 1.

### Statistical analysis

Animals were randomly assigned to experimental groups, time points or traumatic/sham procedures. Mice showing signs of severe distress after awakening from TBI were excluded from analyses. Investigators were blinded to group allocation during data collection and analysis. All statistical analyses were performed using Prism software (GraphPad Software, San Diego, CA) and presented as mean ± standard deviations (SD).

Statistical analyses were performed using Student's *t*-test or Mann Whitney test (when the data were not normally distributed). Two-Way mixed-design ANOVA (genotype x trial) were used in MWM. In the TBI experiments the statistical analyses were performed using Two-Way ANOVA (genotype x treatment) to compare the effects of TBI in Control and p21 cKO mice. Whenever appropriate, post-hoc comparison were performed by Fisher’s protected least significant difference (PLSD) test. In the Rotarod test the data were analyzed by three-way ANOVA (genotype x treatment x time). All experimental groups were composed of at least four animals. The level of significance was set at *P < 0.05; **P < 0.01; ***P < 0.001. All data were expressed as mean values ± SEM.

## Results

### p21 cKO mice characterization

Extensive studies of Cdkn1gene, also known as p21, have revealed its role in several biological processes in mammals, but a more comprehensive study based on a conditional knock-out approach of this gene is still lacking. To this aim, a conditional allele deletion of p21 was designed in our lab, with the CRE/lox system. The critical exon 1 was targeted for the modification: two loxp sites where introduced with CrispR/Cas9 technology [52]. Indeed, eliminating this specific exon, the rest of the coding sequence will be putatively out of frame, possibly inducing non-sense mediated decay [53]. Three different experiment sessions were needed to obtain the correct founder (Fig. 1A). In total, we obtain 2 possible breeders with both Loxp sites correctly inserted but only one gave us germ line transmission (GLT). Subsequent F2 after backcrossing with C57Black6/N were used for later analyses.

To target the specific deletion of p21 in adult Neural Stem Cells, the p21^fl/fl^ mouse has been crossed with the Nestin Cre::ER^T2^ mice model. In the Nestin Cre::ER^T2^/p21^fl/fl^ mouse model obtained (herein referred as p21 conditional Knockout, p21 cKO), the injection of Tamoxifen induced the deletion of the p21 gene in the nestin expressing NSCs of the adult neurogenic niches.

In order to verify the effective conditional deletion of p21 in the DG neurogenic niche, we measured by real time PCR the expression of p21 in the DG of p21 cKO mice 5 and 30 days after 5 daily injections of Tamoxifen. It has been previously demonstrated that in the adult DG p21 is expressed both in the NSCs (Nestin^+^) and in the progenitors [54]. Accordingly, we observed a significant reduction (about 30%) of p21 expression at both the time points in comparison to the control group (5 and 30 days post TAM, Mann Whitney Test, *p* < 0.05, Fig. 1D and E). Moreover, we observed a striking increase of expression of cyclin D2 both at 5 and 30-days after injection of TAM (5 days after TAM, Mann Whitney Test, *p* < 0.05, Fig. 1F; 30 days after TAM, Mann Whitney Test, *p* < 0.05, Fig. 1G), a cell cycle regulator which triggers NSCs proliferation and self-renewal. Finally, we detected an increase of cyclinD1 expression in the p21 cKO only at 5-days after TAM (data not shown).

Finally, we investigated the dynamics of the NSCs isolated from the DG of the Ctrl and p21 cKO groups 5 days after TAM. The data demonstrate a significant decrease of p21 expression in the neurospheres (composed by a mix of NSCs and progenitor cells) isolated from DG of p21 cKO mice (Mann Whitney Test, *p* < 0.01, Fig. 1H). In order to verify the deletion of p21 protein in NSCs we dissociated and plated the neurospheres and performed an immunofluorescence against p21 and Nestin protein. Our data demonstrate that in neurospheres derived from p21 cKO mice there is a significant decrease of p21 (Mann Whitney Test, *p* < 0.001, Fig. 1I, L), an increase of NSCs (Nestin^+^ cells: Mann Whitney Test, *p* < 0.01, Fig. 1J, L) and a total absence of p21 in the nucleus of NSCs (p21^+^ Nestin^+^ cells, Mann Whitney Test, *p* < 0.001, Fig. 1K, L). NSCs neurosphere assay showed a significant increase in the primary cell population forming the DG-derived neurosphere of p21cKO (Mann Whitney Test, *p* < 0.05, Fig. 1M). Finally, we detected an increase in secondary neurosphere number and cell expansion in p21 cKO (for both parameters, Mann Whitney Test, *p* < 0.01, Fig. 1N, O) confirming the conditional deletion of p21 in Nestin^+^ cells lead to a significant activation and proliferative capacity in NSCs.

These results demonstrate that TAM administration induces the deletion of p21 in Nestin-expressing NSCs, and that this event triggers an activation and expansion of hippocampal dentate gyrus NSCs.

On the basis of these data, we aimed at evaluating how the conditional deletion of p21 in NSCs could influence the process of adult neurogenesis in physiological conditions and during the post-traumatic neuro-regenerative response.

### Conditional deletion of p21 enhances activation and expansion of NSCs

**Fig. 2:**
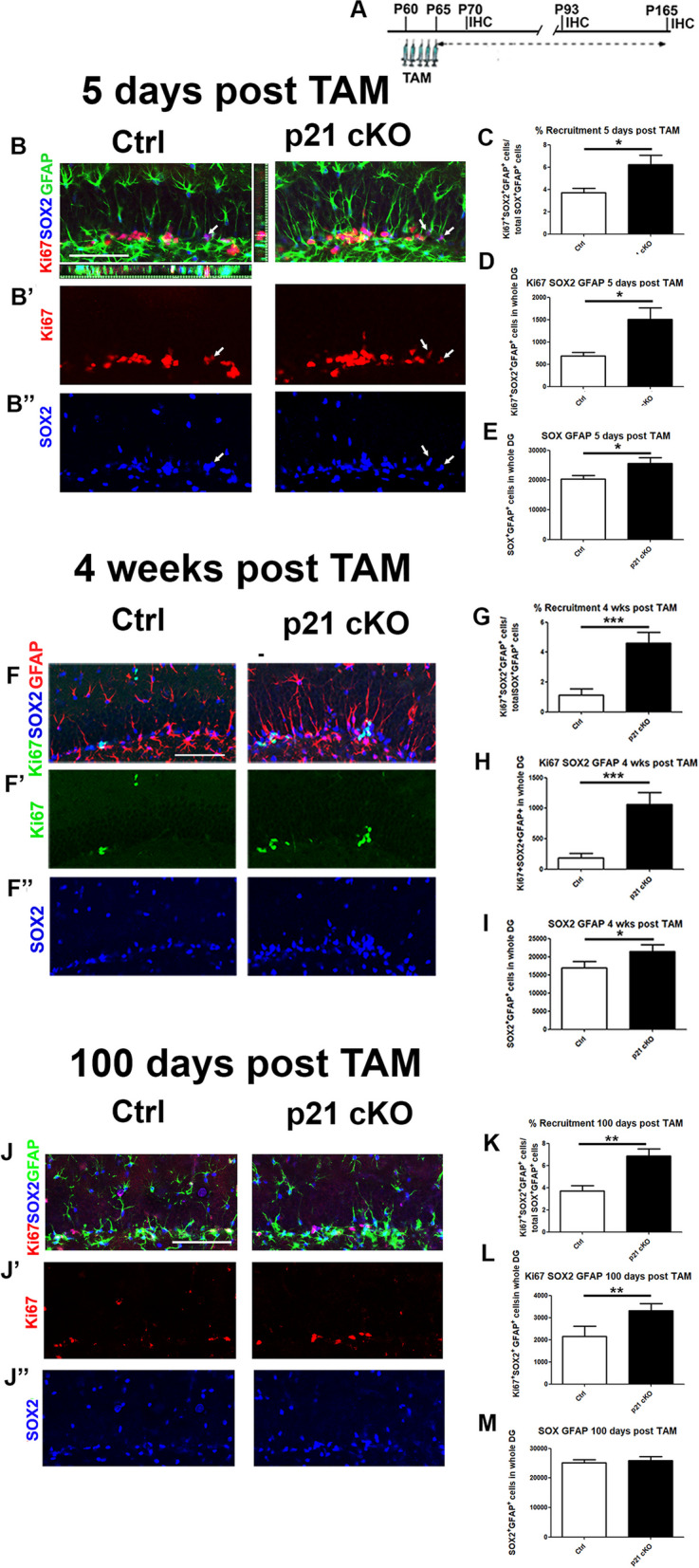
Deletion of p21 in NSCs induces a long-lasting expansion of NSCs pool.** A** Schematic timeline of the experimental procedure. Control and p21 cKO mice were injected with TAM for 5 days and sacrificed 5, 28 and 100 days after the last administration of TAM. **B-B’’** Representative fluorescence confocal images showing the increased recruitment and proliferation of KI67^+^ (red)/SOX2^+^(blue)/GFAP^+^(green) NSCs in the DG of p21 cKO mice respect to the Control group at 5 days after TAM injections. Arrows indicate the NSCs expressing the three specific markers. (B) Representative orthogonal view showing co-localization of KI67^+^ SOX2^+^GFAP^+^ cells in zx and zy axes. **C–E** Graphs illustrating the increased recruitment (ratio between Ki67^+^/SOX2^+^/GFAP^+^ cells/SOX2^+^GFAP^+^ total cells, C), proliferation (number of Ki67^+^/SOX2^+^/GFAP^+^ cells, D) and pool expansion (number of SOX2^+^/GFAP^+^ cells, E) in the p21 cKO at 5 days after TAM. **F–F’’** Representative confocal images describing the enhancement of recruitment and proliferation of KI67^+^ (green)/SOX2^+^(blue)/GFAP^+^(red) NSCs in the DG of p21 cKO mice respect to the Control group 28 days after TAM injections. **G–I** Graphs showing the increment of recruitment (**G**), proliferation (**H**) and pool expansion (**I**) in the p21 cKO respect to the Control mice at 28 days after TAM injections. **J–J’’** 100 days after TAM injections the confocal images demonstrate the increase rate of NSCs (identified by the marker Ki67 red, SOX2 blue and GFAP green) recruitment and proliferation in the DG of p21 cKO mice. **K–M** The graphs show in the p21 cKO mice an increase of NSCs activation (**K**) and proliferation (**L**), without an expansion of NSCs pool (**M**). Statistical significance: *p < 0.05, **p < 0.01, ***p < 0.001, Mann Whitney Test. Scale bar 100 µm. Confocal images magnification 20x. N = 5 animals/group

The main role played by p21 in adult neurogenic niches is to keep stem cells in quiescence. We first evaluated how conditional deletion of p21 affected the activation and proliferative dynamics of adult NSC (aNSCs) at the different time-points (Fig. 2A). The aNSC sub-population was identified through the co-localization of the specific markers GFAP and SOX2 [32]. 5 days after treatment with TAM there is a strong increase in the activation of quiescent aNSCs, as measured by the ratio between the number of proliferating aNSCs (Ki67^+^ SOX^+^ GFAP^+^ cells) and the total number of cells (SOX^+^ GFAP^+^ cells; Mann Whitney Test, *p* < 0.05, Fig. 2B–B’’, C). The rise in aNSCs recruitment in p21 cKO mice is followed by an increase in the number of proliferating aNSCs (Ki67^+^ SOX^+^ GFAP^+^ cells; *p* < 0.05, Fig. 2B, D) and an expansion of the aNSCs pool (SOX^+^ GFAP^+^ cells; Mann Whitney Test, *p* < 0.05, Fig. 2B, B’’, E), compared to the control mouse.

4 weeks and 100 days after TAM injections, we still detected in the p21 cKO mice a significant enhancement in aNSCs recruitment (4 weeks and 100 days; Mann Whitney Test, *p* < 0.01, Fig. 2 F, G, J, K) and proliferation (4 weeks and 100 days; *p* < 0.05, Fig. 2F, H, J, L), leading to a significant expansion of the NSCs pool in the DG of p21 cKO mice at 4 weeks after TAM injections (Mann Whitney Test, *p* < 0.05, Fig. 2F, F’’, I), while 100 days after TAM the NSCs pool size of p21 cKO mice was returned to the control values (Fig. 2J, M).

Collectively, these data demonstrate that p21 deletion in NSCs stimulates a potent activation and proliferation of these cells, which lead to a long-lasting expansion of the pool without causing NSCs depletion over time.

### p21 knockout in NSCs triggers a long-term increase in neuroblasts and proliferation

**Fig. 3:**
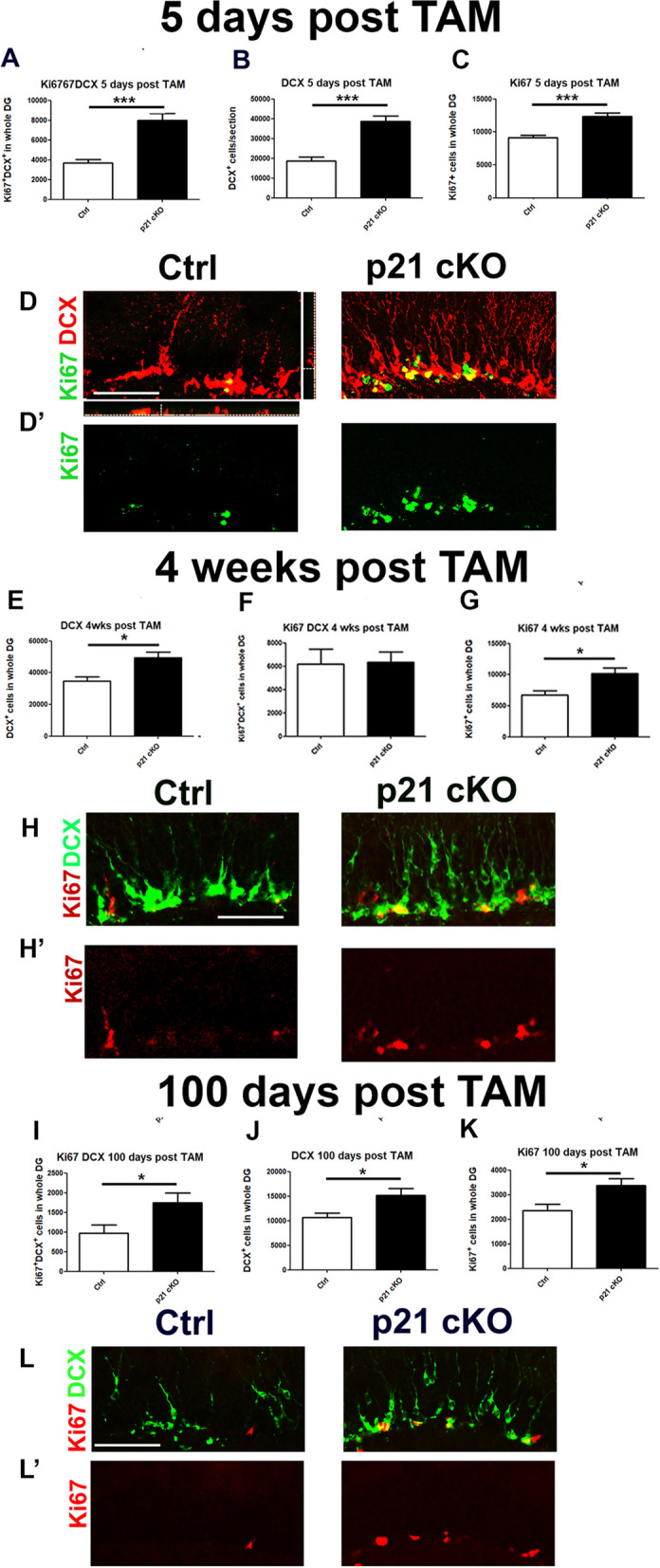
Deletion of p21 in aNSCs induces long-lasting increase of number of neural progenitors (**A**–**B**). Graphs illustrate in the DG of p21 cKO mice an increase in the number of proliferating neuroblasts (Ki67 + /DCX + cells, **A**), leading to an enhancement of total neuroblasts (DCX + cells, **B**) 5 days after TAM injections. **C** Graph showing the increased total proliferation (Ki67^+^ cells) in the DG of p21 cKO mice 5 days after TAM injections **D**–**D’**. Representative confocal images showing that in the DG of p21 cKO group there is a significant increase of DCX^+^ (red) and Ki67^+^ (green) neural progenitors. **D** Representative orthogonal view showing co-localization of KI67^+^ DCX^+^ cells in zx and zy axes. **E–G** The graphs indicate that although no increase in Ki67 + DCX + cells is observed (**E**), the number of DCX^+^ (**F**) and Ki67^+^ (**G**) cells is significantly higher in the DG of p21 cKO mice respect to the Control 28 days after TAM. **H–H’** Representative images indicating the increased number of DCX + neuroblasts (green) and proliferating cells (red) in the DG of p21 cKO mice respect to the Control 28 days after TAM injections. **I**–**K** Graphs describing that 100 days from TAM injection it is still possible to observe a significant increase of proliferating neuroblasts (Ki67^+^/DCX^+^ cells, I), of neuroblasts pool (DCX^+^ cells, J) and of proliferation (Ki67^+^ cells, K) in the p21 cKO mice. **L**–**L’** Representative confocal images showing the increased number of DCX^+^ neuroblasts (green) and proliferating cells (red) in the DG of p21 cKO mice respect to the Control 28 days after TAM injections. Statistical significance: *p < 0.05, **p < 0.01, ***p < 0.001, Mann Whitney Test. Scale bar 100 µm. Confocal images magnification 20x. N = 5 animals/group

The next step was to evaluate how the expansion of the NSC pool in p21 cKO mice could affect the proliferative and differentiation fate of NSC-derived neural progenitor. The analysis of the p21 cKO-induced modulation of DG neuroblasts was carried out by using the specific marker DCX. Our data demonstrate that p21 conditional deletion induced an enhancement of the proliferation of neuroblasts expressing DCX at 5 and 100 days after TAM injections (KI67^+^DCX^+^; 5 days, Mann Whitney Test, *p* < 0.001, Fig. 3A, D; 100 days, Mann Whitney Test, *p* < 0.05, Fig. 3I, L). This event triggers a significant increase in the neural progenitor pool at all the time point examined (DCX^+^: 5, 28 and 100 days after TAM injections; Mann Whitney Test, *p* < 0.05, Fig. 3B, D, F, H, J, L).

Also, the analysis of cell proliferation reveals a stable significant increase of the total number of Ki67^+^ cells in the of DG of p21 cKO compared to the control (5 days after TAM injections: Mann Whitney Test, *p* < 0.001, Fig. 3C, D’; 28 and 100 days after TAM injections, Mann Whitney Test, *p* < 0.05, Fig. 3G, H’, K, L’).

These results show that the effect of p21 deletion in aNSCs triggers a significant increase of proliferative activity in neural and intermediate progenitor cells.

### p21 knockout in NSCs induces a long-term increase of newborn neurons

**Fig. 4:**
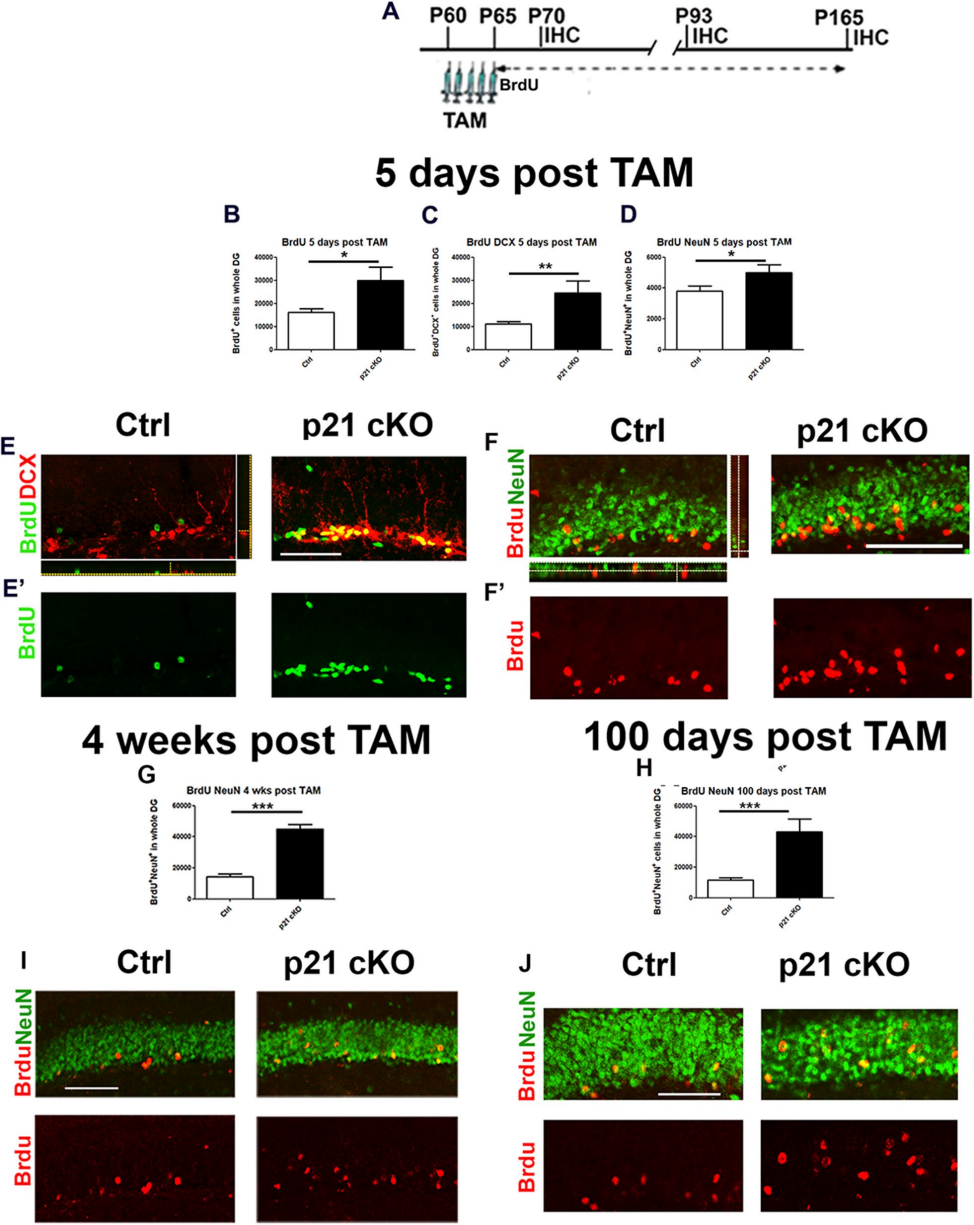
Deletion of p21 in NSCs induces a long-lasting enhancement of neurogenesis. **A** Schematic timeline of the experimental procedure. Control and p21 cKO mice were injected with for 5 days TAM, followed by 5-daily injections of BrdU and sacrificed 5, 28 and 100 days after the last administration of TAM. **B**–**D** Graphs indicating that at 5 days following TAM injections, in the DG of p21 cKO there is a significant increase BrdU^+^ cells (**B**), co-expressing both DCX (**C**) and the marker of mature neuron NeuN (**D**). **E**–**E**’ Representative confocal images showing the increased total number of BrdU^+^ (red) and BrdU^+^DCX^+^ (green and red) neuroblasts in the p21 cKO mice. **E** Representative orthogonal view showing co-localization of BrdU^+^ DCX^+^ cells in zx and zy axes. **F’-F** Representative confocal pictures showing the enhanced total number of BrdU^+^ (red) NeuN^+^ (green) newborn neurons in the p21 cKO mice. **F** Representative orthogonal view showing co-localization of BrdU^+^ Neun^+^ cells in zx and zy axes. **G**, **H** Graphs showing the enhanced number of new mature neurons (BrdU^+^/NeuN^+^ cells) in the DG of p21 cKO mice both 28 (**G**) and 100 (**H**) days after TAM injections. **I**–**I’ and J**–**J’)** Representative images indicating the significant increment of BrdU^+^/NeuN^+^ (red and green, respectively) in the DG of p21 cKO mice both 28 (**I**–**I**’) and 100 (**J**–**J**’) days after TAM. Statistical significance: *p < 0.05, **p < 0.01, ***p < 0.001, Mann Whitney Test. Scale bar 100 µm. Confocal images magnification 20x. N = 5 animals/group

Finally, we wanted to investigate the maturation and survival rate of the new neurons originating in the 5 days following the administration of TAM. To this end we injected the animals for 5 days with BrdU and measured the number of BrdU^+^ cells in the DG at 5, 28 and 100 days after TAM injections (Fig. 4 A). At the first time point we found an overall increase in the total number of BrdU^+^ cells in p21 cKO mice, compared to Control mice (Mann Whitney Test, *p* < 0.05, Fig. 4B, E’), in term of newly generated progenitors at early (BrdU^+^ DCX^+^ cells; Mann Whitney Test, *p* < 0.01, Fig. 4C, E) and at later stage of differentiation (BrdU^+^ NeuN^+^; Mann Whitney Test, *p* < 0.05, Fig. 4D, F’). At 28 and 100 days’ post TAM our data reveals a significant enhancement of BrdU^+^/NeuN^+^ newborn neurons in the DG of p21 cKO in comparison with the Control group (28 and 100 days after TAM injections: Mann Whitney Test, *p* < 0.05, Fig. 4G–J).

These data clearly indicate that the expansion of aNSCs elicited by p21 deletion causes a consistent enhancement of adult hippocampal neurogenesis.

### Effect of conditional p21 deletion on *hippocampus*-dependent behavioural tasks

We next asked whether the powerful proneurogenic action exerted by the conditional deletion of the p21 gene could, to some extent, modify hippocampal neurogenesis-dependent learning and memory abilities. To this end, we performed two hippocampus-dependent behavioural tasks, the Morris Water Maze (MWM) and Novel Object Recognition (NOR); these tests were followed by the expression analysis of the immediate early gene c-Fos in order to evaluate the functional activation of DG neurons. MWM is one of the most frequently used tests to evaluate episodic-like learning and memory, while NOR is the benchmark test of recognition learning and memory in rodents. Both tests are dependent on the integrity of the hippocampus and might reflect changes in neurogenesis. These tests were performed 8 weeks after TAM administration, in 4-month-old mice when hippocampal neurogenesis wanes [55]. However, it has been widely demonstrated that even at this age, adult neurogenesis-dependent cognitive performances are highly efficient and modulable [56, 57].

One day before the above described tasks, we subjected the mice to Open Field (OF) and Elevated Plus Maze (EPM) tests, two behavioural tasks that measure locomotor/exploratory activity and anxiety levels of mice, respectively, in order to exclude that p21 deletion might alter motor skills or mood phenotype of p21 cKO mice. OF data showed no significant difference between the two experimental groups in both motor and exploratory activity (The graphs relative to this mouse skills are shown in Additional file 2: Fig. 3A and B), and in the state of anxiety, as measured by the amount of time spent by the mice in the peripheral squares compared to the central ones (The results are present in Additional file 2: Fig. 3C and D).

In the EPM test, our results clearly evidenced that the two groups did not show significant differences with respect to the time spent in the closed or open arms (The histograms relative to these results are shown in Additional file 2: Fig. 3E, F). Thus, we can conclude that the conditional inactivation of the p21 gene does not induce states of or hypo- and hyper-activity and/or anxiety in mice.

**Fig. 5:**
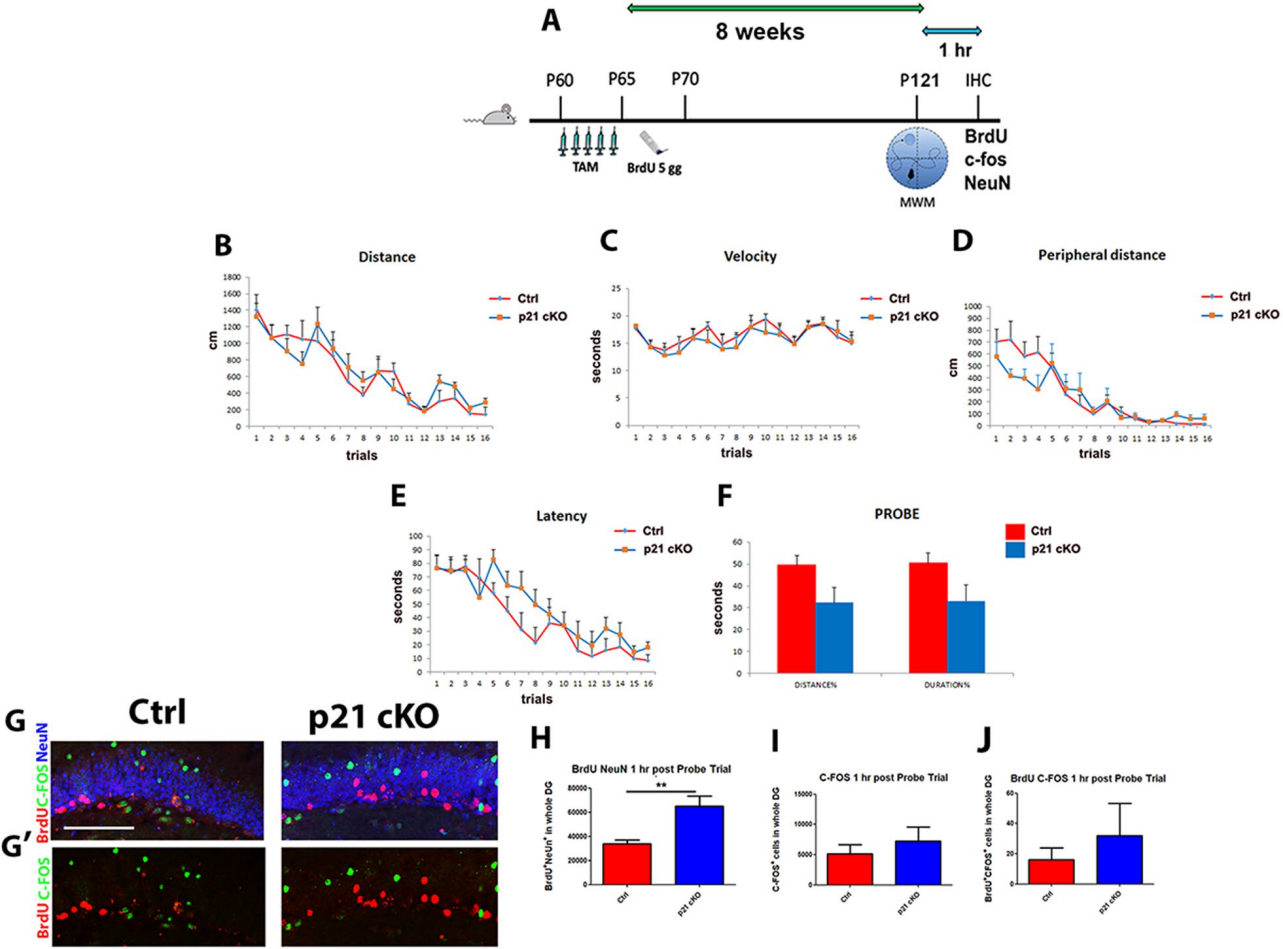
Deletion of p21 in NSCs has no effect on Morris Water Maze. **A** Schematic timeline of the experimental procedure. 2 months-old control and p21 cKO mice were injected with TAM for 5 days, followed by 5 injections of BrdU. 10 weeks after TAM administration mice were subjected to Water Maze behavioural task. 1 h after the end of the test animals were sacrificed and analyzed for the quantification of newborn neurons recruited to the hippocampal neural circuits **B**–**F** Distance (**B**, *genotype* effect: F_1,17_ = 0.83, *p* = 0.37; *trial* effect: F_15,255_ = 12.30, *p* < 0.0000001; *genotype x trial* effect: F_15,255_ = 0.65, *p* = 0.83), velocity (**C**, *genotype* effect: F_1,17_ = 0.40, *p* = 0.54; *trial* effect: F_15,255_ = 4.56, *p* < 0.0000001; *genotype x trial* effect: F_15,255_ = 0.35, *p* = 0.99), latency (**D**, *genotype* effect: F_1,17_ = 3.36, *p* = 0.08, *trial* effect: F_15,255_ = 19.23, *p* < 0.0000001; *genotype x trial* effect: F_15,255_ = 1.25, *p* = 0.23), and percentage of peripheral distance (**E**, *genotype* effect: F_1,17_ = 0.06, *p* = 0.81; *trial* effect: F_15,255_ = 16.98, *p* < 0.0000001; *genotype x trial* effect: F_15,255_ = 1.29, *p* = 0.21, Two-Way mixed-design ANOVA) during the Place phase of the Morris Water Maze (MWM); percentage of distance swum and time spent in the previously rewarded quadrant during the Probe trial (**F**, t-test, *p* > 0.05). **G**–**G’** Representative confocal images illustrating the increased BrdU^+^/NeuN^+^ cells in p21 cKO mice in comparison with the Control mice. **H**–**J** The graphs show the significant increase of BrdU^+^/NeuN^+^ cells in the p21 cKO mice (H), while we did not observe any difference in the number of c-Fos^+^/NeuN^+^ (**I**) and BrdU^+^/c-Fos^+^/NeuN^+^ (**J**) cells between the two groups of animals. Statistical significance **p < 0.01, Mann Whitney Test. Scale bar 100 µm. Confocal images magnification 20x. N = 12 Ctrl and 8 p21 cKO mice

In order to verify if the increase in neurogenesis induced by the conditional deletion of p21 produced an improvement in navigation and spatial learning and memory, control mice and p21 cKO mice were tested in the MWM at 8 weeks after the last injection of Tamoxifen (Fig. 5A). During the Place phase, the total distance travelled and the swimming velocity to reach the platform did not differ between the two experimental groups (Fig. 5B, C). Furthermore, both groups showed a progressive reduction in latency to find the platform as sessions went by, with no significant difference observed between the two groups (Fig. 5D). Moreover, p21 cKO mice swam the same percentage of peripheral distance of the Control group (Fig. 5E). In the Probe trial, the percentage of distance and time spent in the previously rewarded quadrant did not show significant differences between the two groups (Fig. 5F). Taken together, these results indicate that p21 cKO mice exhibit spatial learning and memory capacity comparable to control.

To verify the functional recruitment rate of newborn neurons following the behavioural test, mice were sacrificed 1 h after the end of the probe trial and analyzed for the immune-detection of BrdU (administered 8 weeks before), NeuN and c-Fos (Fig. 5A). We found that the total number of BrdU^+^/NeuN^+^ newborn neurons in the DG was significantly higher in p21 cKO mice compared to Control mice (Mann Whitney Test, *p* < 0.01, Fig. 5G, H). Despite that, the total number of activated neurons (c-Fos^+^ cells), as well as the rate of new functionally recruited neurons (ratio between NeuN^+^/BrdU^+^/c-Fos^+^ and NeuN^+^/c-Fos^+^ total cells), remains unchanged between Control and p21 cKO mice *p* > 0.5, Fig. 5G, I and J). These data indicate that increased neurogenesis induced by p21 deletion was not followed by an increase in functionally activated newborn neurons, and therefore did not contribute to improve spatial memory skills.

**Fig. 6:**
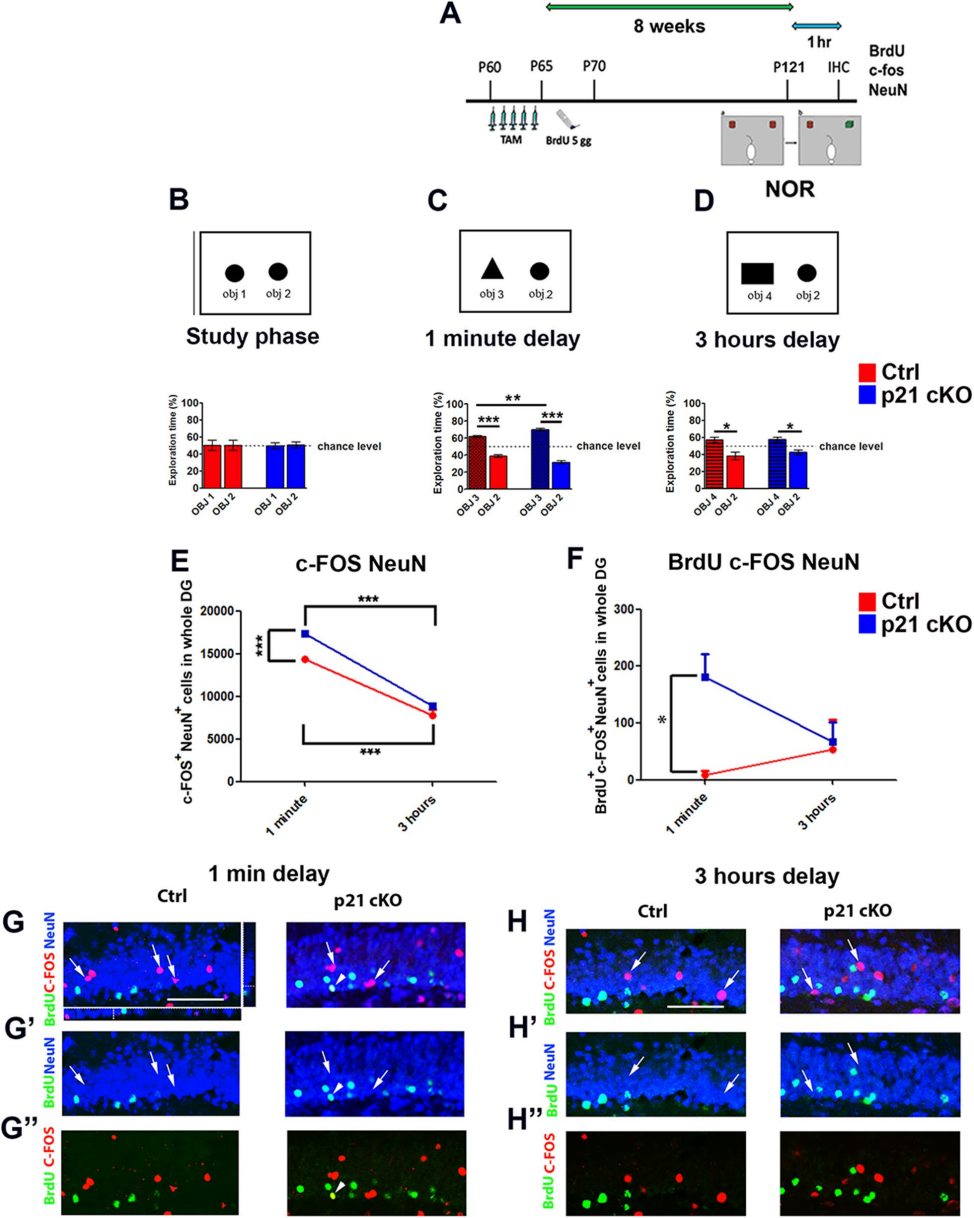
Deletion of p21 in aNSCs increase working memory in NOR. **A** Schematic timeline of the experimental procedure. 2 months-old Control and p21 cKO mice were injected with TAM for 5 days, followed by 5 injections of BrdU. 8 weeks after TAM injections, the mice were subjected to then NOR behavioural task. One hour after the end of the test, the animals were sacrificed and analyzed for the quantification of newborn neurons recruited to the hippocampal neural circuits. **B** Diagram and graph relating to the study phase showing how the Control and p21 cKO mice showed no preference for two identical objects 1 and 2. **C** The graph indicates that 1 min after the study phase both the animal group show a preference for the new object 3, with a significantly higher performance in the p21 cKO mice. **D** In the 3-h delay task the percentage of time spent with the new object 4 is significantly higher respect to the familiar object 1 in both the groups. **E–F** The graphs illustrate the enhancement of c-Fos^+^/NeuN^+^ neurons (**E**) and BrdU^+^/c-Fos^+^/NeuN^+^ (green, red and blue cells, **F**) activated newborn neurons in the DG of p21 cKO mice only in the 1-min delay interval. **G**–**G’’** Representative confocal images illustrating the increased c-Fos^+^/NeuN^+^ cells (red/blue) and BrdU^+^/c-Fos^+^/NeuN^+^ cells (green, red and blue) in p21 cKO mice at 1-min interval. **G** Representative orthogonal view showing co-localization of BrdU^+^, c-Fos^+^ and NeuN^+^ cells in zx and zy axes. Arrows indicate cells double labelled for c-Fos and NeuN antibodies. Arrowhead show cells positive for BrdU, c-Fos and NeuN. **H–H’’** Representative confocal images illustrating the comparable number of c-Fos^+^/NeuN^+^ cells (red/blue) and BrdU^+^/c-Fos^+^/NeuN^+^ cells (green, red and blue) between Control and p21 cKO mice at 3-h.interval. Statistical significance: *p < 0.05, **p < 0.01, ***p < 0.001, Mann Whitney Test. Scale bar 100 µm. Confocal images magnification 20x. Behavioural analysis N = 12 animals/group. Immunofluorescence analysis, N = 5 animals/group

To further analyze the functional effects of p21 deletion on recognition abilities, p21 cKO and WT mice underwent to Novel Object Recognition (NOR) task 8 weeks after, that measures the object-related associative memory utilizing the innate tendency of rodents to explore the novel aspects of the environment or that takes advantage of the rodent's spontaneous preference to explore novel objects relative to familiar ones (Fig. 6A). Mice were initially exposed to two copies of the same object for 10 min and, as expected, they showed no preference for one of them (study phase, Fig. 6B). To explore the possibility that p21 deletion could alter the ability of recognizing a new object, mice were after exposed to a copy of familiar object (object 2) and to a new object (object 3) in a short delay interval (1- minute retention). Both p21 cKO and WT showed a greater preference toward the new compared to the familiar object (Mann Whitney Test, *p* < 0.001, Fig. 6C). However, p21 cKO mice displayed a significantly higher preference for the new object compared to WT (*p* < 0.05, Fig. 6C), suggesting a putative enhancement in working memory. 3 h later, mice were exposed again to a new object (object 4) and a familiar one (object 2) and both the experimental groups displayed a higher percentage of exploration time for the new object 4 (Fig. 6D), without any difference between control and p21 cKO mice. Immunostaining performed 1 h after the 1-min delay interval discrimination task clearly shows in the DG of the p21 cKO group a significant increase of functionally activated neurons (c-Fos^+^/NeuN^+^ cells; Mann Whitney Test, *p* < 0.001, Fig. 6E, G–G’’) and finally of new neurons recruited in memory circuit (BrdU^+^/c-Fos^+^/NeuN^+^, Mann Whitney Test, *p* < 0.05, Fig. 6F, G–G’’). These data strongly suggest a putative contribution of newly-generated neurons in improving the working memory skills of p21 cKO mice. The data obtained after the second exposure of the objects indicate a striking drop of activated neuros (c-Fos^+^/NeuN^+^ cells) (Mann Whitney Test, *p* < 0.001, Fig. 6E, H, H’) and a decrease of functionally recruited new neurons (BrdU^+^/c-Fos^+^/NeuN^+^ cells, Fig. 6F, H, H’’) in the DG of p21 cKO mice. Noteworthy, at this time-point we showed a marked decrease in the number of activated neurons (c-Fos^+^/NeuN^+^ cells,) in both groups, differently to what was observed in the first time-point (Mann Whitney Test, *p* < 0.001, Fig. 6E, G, H). These data suggest that the enhanced neurogenesis dependent on p21 deletion might induce an improvement in the ability of mice to better discriminate a new object 1 min after object habituation, probably reflecting an increase of the working memory skills.

### Post-traumatic response of the NSCs in p21 cKO and control mice

**Fig. 7:**
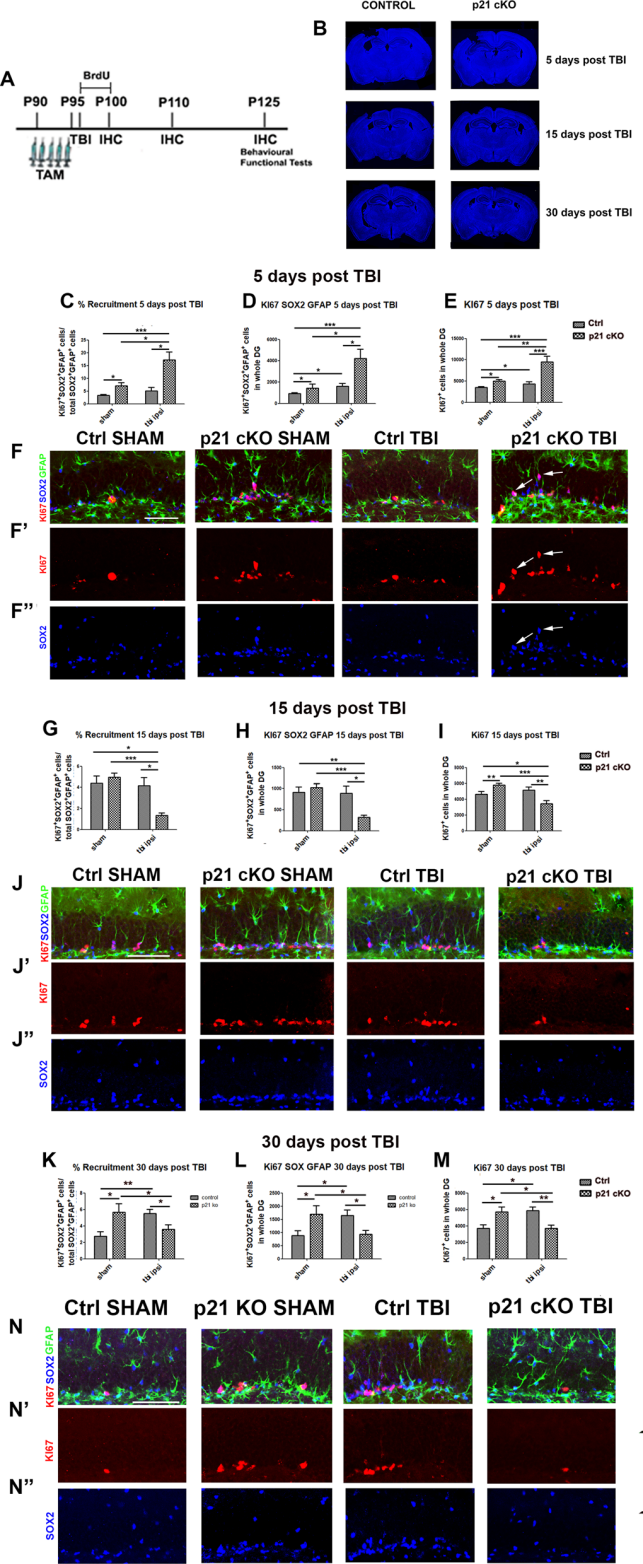
Effect of deletion of p21 in NSCs during post-traumatic neurogenic response. **A** Schematic timeline of the experimental procedure. 3 months-old control and p21 cKO mice were injected with TAM for 5 days. The day after mice were subjected to CCI (TBI groups) or surgical procedure (SHAM groups) and injected with 5 daily injections of BrdU. The 4 groups of mice were analyzed at 5, 15 and 30 days after CCI. Motor coordination analysis (Rotarod) was carried out at 5, 15 and 30 days after CCI, while spontaneous behavior and locomotor activity (Open Field) and cognitive task (Open Field and NOR) were performed 30 days after CCI. **B** Representative images showing the cortical damage induced by CCI at the different time point analyzed. **C–E** Graphs showing the large increment of NSCs recruitment rate (**C,** genotype x TBI interaction: F_(1,30)_ = 6,97, *p* < 0.05, followed by Bonferroni post-test, cKO TBI vs Ctrl SHAM *p* < 0.001, vs cKO SHAM and Ctrl TBI *p* < 0.05), aNSCs proliferation (**D,** genotype x TBI interaction: F_(1,30)_ = 5.96, *p* < 0.05, followed by Bonferroni post-test, cKO TBI vs Ctrl SHAM, cKO SHAM and Ctrl TBI *p* < 0.001) and total proliferation (**E,** genotype x TBI interaction: F_(1,76)_ = 7.38, *p* < 0.01, followed by Bonferroni post-test, cKO TBI vs Ctrl SHAM, cKO SHAM and Ctrl TBI *p* < 0.001,) in the ipsi-lateral DG of p21 cKO TBI mice respect to the other experimental groups 5 days after TBI. **F–F’’** Representative images illustrating the increase in the ipsi-lateral DG of p21 cKO TBI of proliferating NSCs positive for Ki67 (red), GFAP (green) and SOX2 (blue) antibodies. **G–I** After 15 days from TBI, we observe a dramatic drop in the in the ipsi-lateral DG of p21 cKO TBI of NSCs activation (**G**, genotype x TBI interaction: F_(1,39)_ = 5.9 *p* < 0.05, followed by Bonferroni post-test, cKO TBI vs Ctrl SHAM and Ctrl TBI *p* < 0.5, vs cKO SHAM and *p* < 0.001,), NSCs proliferation (**H**, genotype x TBI interaction: F_(1,39_) = 5.9, *p* < 0.05, followed by Bonferroni post-test, cKO TBI vs Ctrl SHAM, cKO SHAM *p* < 0.001 and Ctrl TBI *p* < 0.05) and total proliferation (**I**, genotype x TBI interaction: F_(1,93)_ = 17.11 *p* < 0.001, followed by Bonferroni post-test, cKO TBI vs cKO SHAM and vs Ctrl TBI *p* < 0.05,) respect to the other mice groups. **J–J’’** In these images it is possible to observe the strong decline of proliferating NSCs (Ki67^+^ red/GFAP + green/SOX2^+^ blue cells, **J**) as well as the decrease of total proliferation (Ki67^+^ red, cells, I’) in the ipsi-lateral DG of p21 cKO TBI mice. **K–M** The graphs show the decrease of NSCs recruitment (**K,** genotype x TBI interaction: F_(1,34)_ = 9.8 *p* < 0.01, followed by Bonferroni post-test, cKO TBI vs cKO SHAM and vs Ctrl TBI *p* < 0.05), the NSCs proliferation (**L**, genotype x TBI interaction: F_(1,34)_ = 10.4 *p* < 0.01, followed by Bonferroni post-test, cKO TBI vs cKO SHAM and vs Ctrl TBI *p* < 0.05) and the total proliferation (**M**, genotype x TBI interaction: F_(1,33)_ = 17 *p* < 0.001, followed by Bonferroni post-test, cKO TBI vs cKO SHAM *p* < 0.05 and vs Ctrl TBI *p* < 0.01) in the ipsi-lateral DG of p21 cKO TBI respect to the p21 cKO SHAM and Ctrl TBI mice as well as the significant increase of the above parameters observed in the ipsi-lateral DG of Ctrl TBI mice respect their SHAM group. **N–N’’** Representative images illustrating the decrease in the p21 cKO TBI mice and the increase in the Ctrl TBI mice of proliferating NSCs positive for Ki67 (red), GFAP (green) and SOX2 (blue) antibodies. Statistical significance: *p < 0.05, **p < 0.01, ***p < 0.001. Two-Way ANOVA analysis, by Bonferroni post hoc tests. Scale bar 100 µm. Confocal images magnification 20x. N = 5 animals/group

Increasing evidences suggested that, following Traumatic Brain Injury (TBI), neural stem/progenitor cells may play regenerative and reparative effects in the attempt to repopulate and repair the injured brain [12, 13]. This occurs through a post-traumatic increase in the processes of proliferation, differentiation and maturation of DG newborn neurons. In this study we wanted to verify whether the increased activation of neural stem/progenitor cells in the DG of p21 cKO mice was able to amplify the endogenous neurogenic response following TBI. For this purpose, we analyzed Control and p21 cKO mice at 5, 15 and 30 days after the unilateral CCI (Ctrl TBI and p21 cKO TBI, groups,) and after sham surgery without CCI (Ctrl SHAM and p21 cKO SHAM groups, Fig. 7A). Macroscopic analysis reveals no difference in the post-traumatic tissue repair process between control and p21 cKO mice (Fig. 7B).

As a first step we investigated the NSCs activation and expansion at different times after the trauma. In this regard, we identified this population through the stem markers GFAP and SOX2. At 5-days after the TBI, a Two-Way ANOVA indicates an impressive increase in the recruitment rate (Ki67^+^ SOX^+^ GFAP ^+^ / SOX ^+^ GFAP ^+^ total cells) in the ipsi- and contralateral DG of p21 cKO mice, with respect to the other 3 experimental conditions (TWO-WAY ANOVA: ipsilateral: cKO TBI vs. Ctrl SHAM *p* < 0.001, vs. cKO SHAM and Ctrl TBI *p* < 0.05 Fig. 7C, F–F’’; contralateral: *p* < 0.001, the graphs are shown in Additional file 2: Fig. 4A). This increase caused a significant enhancement of NSCs proliferation (Ki67^+^SOX^+^GFAP^+^ cells) in both hemispheres of p21 cKO mice (TWO-WAY ANOVA: ipsi-lateral: cKO TBI vs. Ctrl SHAM, cKO SHAM and Ctrl TBI *p* < 0.001, Fig. 7D, F; contralateral: *p* < 0.001, the graphs are shown in Additional file 2: Fig. 4B).

The neurogenic analysis performed in the DG 15 and 30-days after TBI revealed a totally different cellular pattern. In fact, in the ipsi- and contralateral DG of the p21cKO mice, we observed a drastic reduction in the aNSCs activation (TWO-WAY ANOVA: Recruitment 15 days: ipsilateral, cKO TBI vs. Ctrl SHAM and Ctrl TBI *p* < 0.05, vs. cKO SHAM and *p* < 0.001, Fig. 7G, J–J’’; contralateral, *p* < 0.001 and *p* < 0.05, the graphs are shown in  Additional file 2: Fig. 4C. Recruitment 30 days: ipsilateral, cKO TBI vs. cKO SHAM and vs. Ctrl TBI *p* < 0.05 cKO TBI vs. cKO SHAM and vs. Ctrl TBI *p* < 0.05, Fig. 7K and N–N’’; contralateral, *p* < 0.05 and *p* < 0.001, the results are shown in Additional file 2: Fig. 4D) and aNSCs proliferation, (TWO-WAY ANOVA: Ki67^+^SOX^+^GFAP^+^ cells 15 days: ipsilateral, ipsi-lateral, cKO TBI vs. Ctrl SHAM, cKO SHAM *p* < 0.001 and Ctrl TBI *p* < 0.05, Fig. 7H, J; contralateral, *p* < 0.001 and *p* < 0.05, the data are present in Additional file 2: Fig. 4E. Ki67^+^SOX^+^GFAP^+^ cells 30 days: ipsi-lateral, cKO TBI vs. cKO SHAM and vs. Ctrl TBI *p* < 0.05, Fig. 7L, N; contralateral, *p* < 0.01 and *p* < 0.001, the graphs are shown in Additional file 2: Fig. 4F) compared to the other experimental groups.

Analysis of total cell proliferation within injured DG showed a strong increase of Ki67^+^ cells in p21 cKO mice at 5 days after the TBI (TWO-WAY ANOVA: ipsilateral, cKO TBI vs. Ctrl SHAM, cKO SHAM and Ctrl TBI *p* < 0.001 cKO TBI vs. Ctrl SHAM, cKO SHAM and Ctrl TBI *p* < 0.001 cKO TBI vs. Ctrl SHAM, cKO SHAM and Ctrl TBI *p* < 0.001, Fig. 7E, F’; contralateral, *p* < 0.001, the results are shown in Additional file 2: Fig. 4G), followed by an evident drop at 15 days (TWO-WAY ANOVA: ipsi-lateral, cKO TBI vs. cKO SHAM and vs. Ctrl TBI *p* < 0.05, Fig. 7I and J’; contralateral, *p* < 0.001 and *p* < 0.01, the dare are represented in Additional file 2: Fig. 4H) and 30 days after TBI (ipsilateral, cKO TBI vs. cKO SHAM *p* < 0.05 and vs. Ctrl TBI *p* < 0.01, Fig. 7M and N’; contralateral, *p* < 0.05 and *p* < 0.01, the results are shown in Additional file 2: Fig. 4I).

In the Ctrl mice TBI induces a significant rise of NSCs activation at 30 days post trauma (ipsilateral, *p* < 0.01, Fig. 7K, N), while an enhancement of NSCs proliferation is detectable at 5 (Ki67^+^ SOX^+^ GFAP^+^ cells; ipsilateral, *p* < 0.05, Fig. 7D, F; contralateral, *p* < 0,001, the results are present in Additional file 2: Fig. 4B) and 30 days post TBI (ipsilateral, *p* < 0.05, Fig. 7K, N). We finally observed that the increased of total proliferation respect to the Control SHAM group becomes evident 30 days post TBI (ipsi- and contralateral, *p* < 0.05, Fig. 7M, N’ and  the data relative of controlateral DG are present in Additional file 2: Fig. 4I).

These data indicate a combined effect of conditional downregulation of p21 and TBI in inducing a rapid and powerful activation and proliferation of NSCs followed by an equally rapid depletion of the pool of stemness in the DG of p21 cKO mice.

### Post-traumatic response of the neural progenitors and newborn neurons in p21 cKO and control mice

**Fig. 8:**
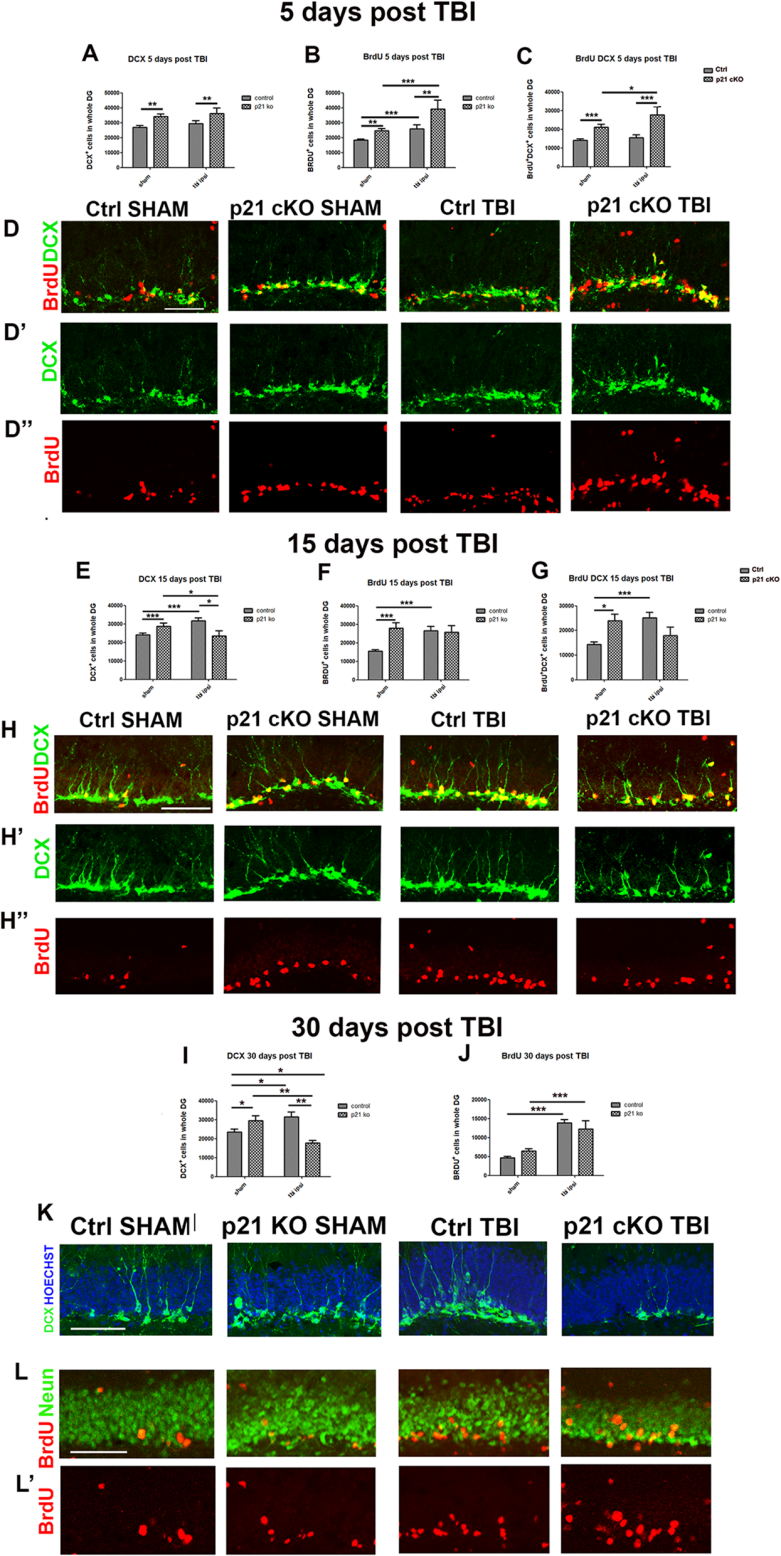
Effect of deletion of p21 in neurogenesis during post-traumatic neurogenic response. **A** Graph illustrating the effect of p21 deletion in the increase of DCX^+^ cells in the ipsi-lateral DG 5 days after TBI. **A**–**C** Graphs showing the effect of p21 deletion in the increase of DCX^+^ cells in the ipsi-lateral DG 5 days after TBI. The data indicates that 5 days after TBI there is a significant increment of DCX^+^ (**A**, genotype effect F_(1,70)_ = 10,12, *p* < 0.01, Fig. 8 A, D’), an increment of BrdU^+^ (**B**, genotype effect: F_(1,37)_ = 10,69, *p* < 0.01, TBI effect: F_(1,37)_ = 13,36, *p* < 0.001,) and BrdU^+^/DCX^+^ cells (**C**, genotype effect: F_(1,35)_ = 27, *p* < 0.001, TBI effect: F_(1,35)_ = 4,4, *p* < 0.05,) in the ipsi-lateral DG of p21 cKO TBI mice respect to the other groups. **D**–**D’’** Representative images showing the increased number of BrdU^+^ (red, **D’’**) and BrdU^+^ (red)/DCX^+^ (green, **D**) newborn progenitor cells in the p21 cKO TBI mice compared to the other groups. **E** Graph showing the increase of DCX^+^ cells in the ipsi-lateral DG of Ctrl TBI group respect to the Ctrl SHAM group and the decrease of DCX^+^ cells in the ipsi-lateral p21 cKO TBI mice in comparison with Ctrl TBI and p21 cKO mice, 15 days after TBI. **E**–**G** Graphs indicating a significant decrease of DCX + cells (**E**, genotype x TBI interaction: F_(1,104)_ = 12,17 *p* < 0.001, followed by Bonferroni post-test, cKO TBI vs Ctrl SHAM *p* < 0.05, vs cKO SHAM *p* < 0.001 and vs Ctrl TBI *p* < 0.01), an increase of BrdU + (**F,** genotype x TBI interaction: F_(186)_ = 6, *p* < 0.05, followed by Bonferroni post-test, cKO TBI vs Ctrl SHAM, cKO SHAM and vs Ctrl TBI *p* < 0.05) and BrdU^+^/DCX^+^ (**G,** genotype x TBI interaction: F_(1,57)_ = 9.3 *p* < 0.01, followed by Bonferroni post-test, cKO TBI vs Ctrl SHAM, cKO SHAM and vs Ctrl TBI *p* < 0.05) cells in the ipsi-lateral DG of Ctrl TBI mice respect their SHAM littermates, 15 days after TBI. **H–H’’** Confocal micrographs showing the enhanced number BrdU^+^ (red, **H’’**) and BrdU^+^ (red)/DCX^+^ (green, **H**) newborn progenitors cells in the Ctrl TBI mice respect to the Ctrl SHAM group, 15 days after TBI. (**I**) Graph showing: 1) the significant drop in the number of DCX^+^ in the ipsi-lateral DG of p21 cKO mice respect to the other experimental condition ((genotype x TBI interaction: F_(1,31)_ = 22 *p* < 0.001, followed by Bonferroni post-test, cKO TBI vs cKO SHAM *p* < 0.05 and vs Ctrl SHAM and Ctrl TBI *p* < 0.01) and 2) the increase of DCX^+^ cells in the ipsi-lateral DG of Ctrl TBI mice respect to the Ctrl SHAM group (ipsi- and contra-lateral: Ctrl TBI vs Ctrl SHAM *p* < 0.05) 30 days after TBI. **J** Graph showing the TBI-effect in the increase of BrdU^+^ cells in the ipsi-lateral DG, 30 days after TBI. **K** Confocal images showing the decreased number of DCX^+^ (green) in the p21 cKO mice and the BrdU^+^ cells enhancement in the Ctrl TBI group respect to the Ctrl SHAM mice. **L** Confocal pictures illustrating the increased number of BrdU^+^ (red)/NeuN^+^ (green) cells in the groups underwent to TBI when compared with the SHAM mice. Statistical significance: *p < 0.05, **p < 0.01, ***p < 0.001. Two-Way ANOVA analysis, by Bonferroni post hoc tests. Scale bar 100 µm. Confocal images magnification 20x. N = 5 animals/group

Study of the post-traumatic dynamics of DCX^+^ neural progenitors revealed a significant genotype effect in the increase of DCX^+^ neuroblasts 5 days after TBI (TWO-WAY ANOVA: ipsi-lateral, *p* < 0.01, Fig. 8A, D’; contralateral, *p* < 0.01, the results are shown in Additional file 2: Fig. 5A). Moreover, our data indicated a drastic reduction of DCX^+^ cells in the p21 cKO DG at 15 days after TBI (TWO-WAY ANOVA: ipsi-lateral, cKO TBI vs. Ctrl SHAM *p* < 0.05, vs. cKO SHAM *p* < 0.001 and vs. Ctrl TBI *p* < 0.01, Fig. 8E and H’; contralateral, *p* < 0.001 and *p* < 0.05, the data are displayed in Additional file 2: Fig. 5B) and 30 days after post TBI (TWO-WAY ANOVA: ipsi-lateral, cKO TBI vs. cKO SHAM *p* < 0.05 and vs. Ctrl SHAM and Ctrl TBI *p* < 0.01, Fig. 8I and K; contralateral, *p* < 0.05, *p* < 0.01 and *p* < 0. 001, the results are present in  Additional file 2: Fig. 5C).

In control mice, TBI induces a significant increase of DCX^+^ neural progenitors in the DG of both hemispheres compared to its SHAM group both at 15 days (ipsi- and contralateral: *p* < 0.001, Fig. 8E, H and the controlateral data are shown in Additional file 2: Fig. 5B) and 30 days after TBI (ipsi- and contralateral, *p* < 0.05, Fig. 8I, K and the controlateral results are shown in Additional file 2: Fig. 5C).

Finally, we investigated the fate of new neurons in control and p21 cKO mice injected with BrdU for 5 days following TBI and analyzed at the same time-points described above, with the aim of verifying how the post-traumatic processes of maturation and survival were affected by the conditional deletion of p21.

Our data demonstrated that at 5 days after TBI there is a significant genotype and TBI effects in the increase in BrdU^+^ cells (TWO-WAY ANOVA: *p* < 0.01 and *p* < 0.001, respectively, Fig. 7B, D, D’’) and BrdU-expressing neuroblasts in the ipsi-lateral hemisphere (BrdU^+^DCX^+^ cells, *p* < 0.001 and *p* < 0.05, respectively, Fig. 8C, D’’), while in the contralateral hemisphere we observed a significant genotype effect in the increase of BrdU^+^ (TWO-WAY ANOVA: *p* < 0.001, the results are present in Additional file 2: Fig. 5D) and BrdU^+^DCX^+^ cells (TWO-WAY ANOVA: *p* < 0.001, the date are represented in Additional file 2: Fig. 5E).

Moreover, in the ipsilateral DG of the p21 cKO group at 15 days after the TBI we observed that the number of newborn neurons (BrdU^+^ cells) and their progenitor component (BrdU^+^ DCX^+^ cells) is not significantly different to that observed in the other groups of mice (BrdU^+^ cells, Fig. 8F, H’; BrdU^+^ DCX^+^ cells, Fig. 8G, H), while 30 days after TBI we detect TBI effect in the number of new mature neurons (TWO-WAY ANOVA: BrdU^+^/NeuN^+^ cells, *p* < 0.001, Fig. 8J, L, L’).

In the Control mice subjected to TBI we observed in the ipsilateral region a constant increase of BrdU + cells compared to the Ctrl SHAM group at all times considered (5, 15 and 30 days’ post TBI, *p* < 0.001, Fig. 8B, D’’, F, H’’, J, L), while there is no increase significant number of new neurons in the contralateral region.

These data suggest that, despite the strong decrease in proliferation and differentiation in the p21 cKO TBI group in the medium to long term, the processes of maturation and survival of the considerable number of new neurons produced in the 5 days following the TBI were not significantly altered.

Taken together, these results clearly indicate that (i) in physiological conditions, there is a gradual increase in adult hippocampal neurogenesis as a response to TBI and (ii) the deletion of p21 causes in the medium-long term a net worsening of hippocampal neurogenic parameters, as a consequence of the excessive pro-neurogenic response occurring immediately after the traumatic injury.

### Functional and cognitive post-traumatic response of the p21 cKO mice

**Fig. 9:**
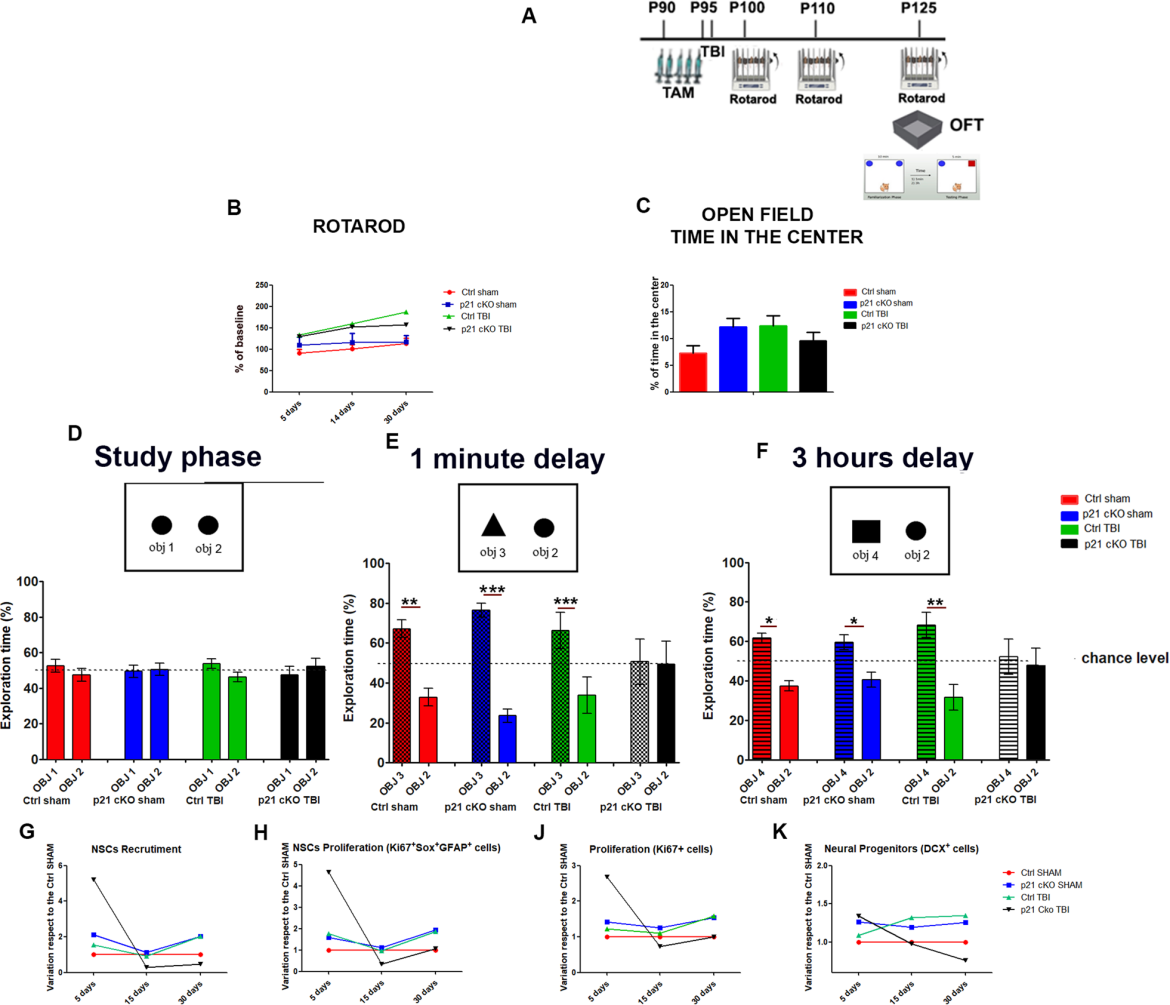
Functional and behavioural outcomes after TBI. **A** Schematic timeline of the experimental procedure, already described in Fig. 7A. **B** Graph showing that TBI does not induce significant differences in the Rotarod test within the 4 experimental groups at the different time-point after lesion (Three-way ANOVA genotype x TBI x time, F _(1,57)_ = 0.9, *p* > 0.05). **C** The graph indicates that the animal of the 4 groups spend comparable amount of time in the Centre of the Open Field (Two-Way ANOVA *p* > 0.05). **D** The graph indicates that all the 4 groups of mice spent a comparable percentage of time with the identical object 1 and 2. **E**, **F** The graphs indicate that the Ctrl SHAM, cKO SHAM and Ctrl TBI display a significantly higher preference towards the new respect to the familiar object at the 1-min interval (t-Test object 3 vs object 2: Ctrl SHAM *p* < 0.01, cKO SHAM *p* < 0.05 and vs Ctrl TBI *p* < 0.001), as well as after 3 h from the study phase (t-Test object 4 vs object 2: Ctrl SHAM *p* < 0.05, cKO SHAM *p* < 0.05 and vs. Ctrl TBI *p* < 0.01). **G-K** Graphs representing the variation rate of NSCs recruitment (G) and proliferation (H) as well as total proliferation (K) observed in the cKO SHAM, Ctrl TBI, cKO TBI mice respect to the Ctrl SHAM group. Statistical significance: *p < 0.05, Mann Whitney Test. N = 12 animals/groups

TBI is known to induce motor, psychological and cognitive deficits, comprising severe impairment in adult hippocampal neurogenesis-dependent learning and memory tasks. To deepen this issue, Control and p21 cKO-animals, were evaluated using comprehensive motor, anxiety and cognitive functional tests through 30 days post injury. Motor coordination and learning were assessed using the Rotarod task, anxiety and explorative behaviour with the Open Field and cognitive skills with the Novel Object Recognition task (Fig. 9A).

In the Rotarod test, pre-injury performance did not reveal any significant differences in the fall latencies between Control and p21 cKO mice. Post-injury comparisons were carried out by recording the time each mouse spent on the rod (latency) as percentage of its baseline latency. Our data show that brain-injured Control and p21 cKO mice did not perform worse in this task than sham-injured mice of the same genotype at 5, 14 and 30 days’ post TBI (Fig. 9B).

The Open Field task carried out 30 days after the TBI did not show any differences between the 4 different experimental groups, suggesting that the brain injury did not alter the brain regions responsible for the motor and exploratory skills, as well as for anxiety or alternatively that at this time point functional reorganization may have occurred (Fig. 9C).

To determine whether the conditional ablation of p21 affected injury-induced cognitive deficits, we performed the Novel Object Recognition 1 month after p21 knockout and TBI. The test was carried out with the same parameters used previously, i.e. presentation of the new object 1 min and of a second new object 3 h after the presentation of the two identical objects. During the study phase we did not observe in the 4 experimental groups any difference in the preference for either of the two explored identical objects (object 1 vs. object 2, Fig. 9D). In the subsequent NOR tasks (1 min and 3 h-delay) TBI induced a clear cognitive impairment in the performance p21 cKO mice. Indeed, this group of mice show a complete deficiency in discriminating new objects both a 1 min and 3 h after the study phase (Fig. 9E, F), suggesting that the declined adult neurogenesis induced by p21 deletion and TBI might negatively impacts on hippocampus-dependent cognitive abilities, in term of working and short-term memory.

## Discussion

Here we demonstrated for the first time that p21 deletion in the aNSCs results in a completely different outcome of adult hippocampal neurogenesis under phisiological or post-traumatic conditions. Indeed, we observed a strong and long-term increase of adult hippocampal neurogenesis in the p21 cKO mice, while TBI triggers a process of early hyper-activation and proliferation of p21 cKO NSCs, followed by their rapid depletion and by a significant decline of adult neurogenesis.

p21 is an essential regulator of the cell cycle that plays a fundamental role in modulating the quiescence/recruitment state of NSCs and in maintaining the homeostasis of the neurogenic niches of the adult brain [30, 33]. It has been previously demonstrated that the expression of p21 in the hippocampal neurogenic niche is confined to the aNSCs and neuroblasts of the DG [54], suggesting its specific role in maintaining aNSCs in quiescence via G1 proliferation arrest [31] and in restraining the expansion of neuroblasts [34, 58]. Numerous studies have explored the neurogenic regulatory role of this gene in vivo through the use of a murine model of constitutive deletion of p21, with contrasting results depending on the different neurogenic niches and developmental stages [33, 34, 37, 54]. In this work we present for the first time new data describing the phenotypic and behavioral effects resulting from the conditional deletion of the p21 gene within adult NSCs in the hippocampal DG. Our data clearly demonstrate that the deletion of p21 in NSCs induces a powerful and long-lasting activation of adult hippocampal neurogenesis, as a result of an initial increase in the recruitment and proliferation of p21-lacking NSCs, leading to a significant enhancement of the proliferation and differentiation of neuroblasts and finally the maturation and survival of mature neurons. These data are in full agreement with previous observations describing how the constitutive deletion of p21 was able to increase the proliferation of new neurons [54] int the DG of adult mice, and broaden its relevance by demonstrating a powerful pro-neurognic long-term effect induced by conditional deletion of p21. One of the main results of our study is represented by the observation of the increased ability of p21-lacking aNSCs to exit from quiescence to undertake the proliferative path without signs of aNSCs depletion even 100 days after TAM injections. Once released from quiescence, the aNSCs can adopt different modalities of division in order to simultaneously allow the integrity of the pool and the generation of diversified progenies [14]. In this regard, different theories tried to explain the fate of aNSCs, some hypothesizing that the DG aNSCs that have left quiescence become rapidly astrocytes after a series of asymmetric neurogenic division without returning to quiescence [59]; others instead suggest a return to quiescence of activated aNSCs in order to preserve the aNSCs pool [60–62]. Our study, although not describing in detail the cell mode division of p21-null aNSCs, is oriented towards the hypothesis that p21-null aNSCs have the capacity for cell expansion in the short- medium- without proliferation impairment. Partial support of this hypothesis is the increase in the expression of cyclin D2, one of the cell cycle genes mainly involved in the proliferative progression of aNSCs [63, 64], in the DG of p21 cKO mice at 7 and 28 days post TAM. In this regard, a decrease in hippocampal adult neurogenesis was observed in the cyclin D2 knock out mice model [65], while the over-expression of the CDK4/cyclin D1 complex in aNSCs induces a powerful pro-neurogenic effect [66]. However, at 100 days post TAM, an increase in the pool of NSCs is no longer observed in the p21 cKO mouse, leading us to hypothesize that the deletion of p21 in aNSCs may induce an increase in symmetric terminal divisions at the expense of asymmetric self-renewing division.

In order to analyze the causal link between the increased neurogenesis in p21 cKO mice and hippocampal-dependent learning and memory abilities, we tested the animals in the Morris Water Maze and Novel Object Recognition, both tests modulated by adult hippocampal neurogenesis [45, 67]. In the present study, we found a discrepancy between the behavioral outputs related to increased hippocampal adult neurogenesis in p21 cKO mice. In fact, the increase in newborn neurons in the p21 cKO mice does not correspond to any improvement in the Morris Water Maze task, while we observed an improvement in working memory ability in the Novel Object Recognition test.

The increased neurogenesis observed in p21 cKO mice at the end of MWM task was not accompanied by an increase in functionally activated newborn neurons in the DG, thus leaving long-term spatial memory performance unaltered. The impact of the modulation of adult hippocampal neurogenesis on the different phases of the MWM is highly variable, as indicated by highly discordant results [67]. One of the factors moslty influencing the behavioral response in the MWM in correlation with neurogenic changes, in both learning (navigation) and in memorization (probe trial) phase, is the age of the new neurons involved. In fact, it has been described that new neurons of about 4 weeks display a greater recruitment capacity in the spatial memory circuits and consequently their deletion [65, 68] or increase [69, 70] produce a strong impact in MWM behavioral responses. Conversely, in the present study the p21 cKO animals were tested in the MWM 8 weeks after TAM injections, a time interval that likely did not allow to show the link between increased neurogenesis and improved abilities in spatial learning and memory, in line with the results obtained in a murine model of enhanced neurogenesis [71].

The data acquired in NOR demonstrate that at the 1-min delay interval, although both experimental groups performed above chance, p21 cKO mice showed an increased preference towards the new respect to the familiar object associated with a strong increase in c-Fos^+^/NeuN^+^ and BrdU^+^/c-Fos^+^/ NeuN^+^ cells in the DG. At the 3-h delay interval, the two experimental groups show a similar performances in the discrimnation of the famialiar objects, indicating that the deletion of p21 does not alter the retention of previously acquired information. These data suggest that: (a) the increase in neurogenesis induced by the deletion of p21 in aNSCs induced a significant improvement in the working memory, a cognitive system dependent on adult hippocampal neurogenesis [71–74], (b) this improvement likely involves the functional contribution of 8-week-old newborn neurons. A similar correlation has been demonstrated recently by two studies which have shown how physical activity in synergy with cognitive training [72] or with the administration of lactate [70] is able to promote an increase in adult hippocampal neurogenesis and a consequent increase in working memory perfomance. The involvement of p21 in such mechanisms has not been taken into consideration, however we have demonstrated that the deletion of p21 is able to largely amplify the pro-neurogenic role of physical activity in the subventricular neurogenic niche [37, 38]. Finally, it has been widely highlighted how various pro-neurogenic factors (aspirin, polyphenols and other nutritional compounds) are able to increase working memory performance [71, 74–76].

The most important result arising from our work, however, is the observation of the dramatic long-term decrease in hippocampal neurogenesis observed in the p21 cKO mice, following TBI. Numerous studies have highlighted how a moderate-severe CCI induces an increase in the rate of hippocampal neurogenesis, with a portion of new neurons able to mature, survive and integrate into hippocampus-dependent memory circuits [74–77]. Our data on one side confirm the pro-neurogenic response following TBI in the Ctr TBI group [78–80], and on the other side highlight for the first time the fundamental role of p21 in finely modulating the activation and proliferation of NSCs in early post-traumatic stages. In fact, the hyper-activation and proliferation of p21-lacking aNSCs observed 5 days after TBI resulted into a rapid depletion of the NSC pool inducing a subsequent dramatic decrease of hippocampal neurogenesis associated with a profound deficit in the NOR perfomance at 30 days post-TBI. Indeed, p21 cKO TBI mice are completely incapable of discriminating the new object both 1 min and 3 h after the study phase, suggesting a role of hippocampal neurogenesis in object recognition memory processes. These results lead us to hypothesize that the expression of p21 within adult NSCs may in some way represent a sort of “compromise” which on the one hand limits the neurogenic potential of NSCs in physiological conditions, and on the other hand favors a correct neuro-reparative response after brain injury. Although further studies will be necessary to gain the molecular understanding of these observations, our study suggests a new fundamental role of p21 in maintaining the quiescence/activation balance of NSCs during the post-traumatic neurogenic response.

Finally, by comparing the dynamics of activation/proliferation of NSCs in Ctrl TBI and p21 cKO SHAM mice, we observed a very similar trend in the increase in NSCs recruitment and proliferation (Fig. 9E, F) as well as in total proliferation (Fig. 9G), in the two groups respect to the Ctrl mice, despite the profound diversity of the pro-neurogenic stimuli, exogenous for the Ctrl TBI group and genetic for the p21 cKO mice. This observation suggests that within the hippocampal neurogenic niche there may exist an common intrinsic mechanism, likely involving p21 expression, able to to keep the activation/proliferation rate of NSCs below a certain threshold in order to maintain the neurogenic potential of aNSCs. Once this threshold is exceeded, as in the case of p21 cKO TBI mice, there is a progressive depletion of the stem cell pool. However, the cellular and molecular processes through which the aNSCs exhaustion might occur are still to be clarified. It has been demonstrated that hyper-proliferation of NSCs triggered by the ablation of quiescence-maintaining gene (i.e. Integrin β1 or Mfg8) or by kainic acid-induced epileptic seizures provokes a rapid consumption of aNSCs pool [24, 81–83]. In this regard Noguchi et al. have recently shown that the deletion of Sonic Hedgeog (Shh) in the mossy fibers of the hippocampus induces a premature and accelerated depletion of the NSCs pool following seizure-induced neurogenesis [84], suggesting a primary role of this factor in maintaining aNSCs and to prevent exhaustion from excessive consumption after seizure.

## Conclusion

Our study identifies for the first time how in a post-traumatic context, p21 plays a pivotal role in mantaining the balance between stem cell quiescence and activity, regulating therefore the rate of neurogenesis and allowing the long-term survival of the aNSCs pool. Therefore, the modulation of p21 in the aNSCs could represent a promising genetic and/or pharmacological tool capable of enhancing the neuroplasticity processes involved in neuro-reparative processes.

## Supplementary Information

**Additional file 1**. Material and methods.

**Additional file 2**. Supplementary Figures and Tables.

## Data Availability

All data generated or analysed during this study are included in this published article (and its supplementary information files).
