# Correction: Essential role of p21^Waf1/Cip1^ in the modulation of post-traumatic hippocampal Neural Stem Cells response

**DOI:** 10.1186/s13287-026-05142-x

**Published:** 2026-06-30

**Authors:** Francesco Chiani, Valentina Mastrorilli, Nicole Marchetti, Andrea Macioce, Chiara Nappi, Georgios Strimpakos, Miriam Pasquini, Alessia Gambadoro, Jonathan Isacco Battistini, Debora Cutuli, Laura Petrosini, Sara Marinelli, Raffaella Scardigli, Stefano Farioli Vecchioli

**Affiliations:** 1 Institute of Biochemistry and Cell Biology, IBBCCNR, Monterotondo, Rome, Italy; 2 PhD Course in Sciences of Nutrition, Aging, Metabolism and Gender Pathologies, Catholic University of Roma, Rome, 00100 Italy; 3 Instituto de Neurosciencias, Universidad Miguel-Hernandez, Alicante, Spain; 4 Department of Psychology, Sapienza University of Rome, Via dei Marsi 78, Rome, 00185 Italy; 5 IRCCS Fondazione Santa Lucia, Via Ardeatina 306, Rome, 00179 Italy; 6 European Brain Research Institute (EBRI), Viale Regine Elena, Rome, 00161 Italy; 7 Institute of Translational Pharmacology, National Research Council, Rome, Italy

**Correction: Stem Cell Research & Therapy (2024) 15:197** 10.1186/s13287-024-03787-0

The following Funding acknowledgement was mistakenly omitted from the original article [1] and is noted ahead:

"This article was funded by the European Union – Next Generation EU, Mission 4 Component 1—PRIN 2022 Project, Title: "Characterization and modulation of “immature” neurons: a potentially exploitable reservoir of non-newly generated cells involved in plasticity of the rodent and human cerebral cortex" code 2022LB4X3N_LS5_PRIN2022, CUP B53D23018760006."
