# Supplementary material for: Essential role of p21Waf1/Cip1 in the modulation of post-traumatic hippocampal Neural Stem Cells response
Source: Stem Cell Res Ther. 2024 Jul 6;15:197. doi: 10.1186/s13287-024-03787-0 (PMC11227726; doi:10.1186/s13287-024-03787-0)
Supplement: Supplementary file 1 — Additional file 1. Material and methods. [file 13287_2024_3787_MOESM1_ESM.docx]

**ADDITIONAL FILES 1**

**MATERIAL AND METHODS**

**GENERATION OF p21^floxed^ MICE**

**Allele design**

After CRE recombination the allele will lead to (Fig.1A [II]) an out of frame transcript downstream, target of non-sense mediated decay. The proximal Loxp (pLoxp) was obtained inserting a 40 bp sequence bearing HindIII recognition site sequence and a standard Loxp sequence (Fig.1A). Pam sequence was destroyed in the donor single strand DNA (pssDNA) to avoid Cas9 recurrent cutting. Each homologous arm of the ssDNA donor template is at 60bp from the putative Cas9 cutting site, and the sgRNA sequence is shown in gray empty arrow and labelled as SgRNA1. The distal loxp site (dLoxp) is placed in the intron 2. As for the pLoxp, a restriction site for KpnI was added and PAM site was modified as in the previous in the donor template (dssDNA) to avoid recurrent cutting of Cas9, and both the arm are 60bp from the predicted Cas9 cutting site. SgRNAs and Cas9 mRNA were in-vitro synthetized as described in (40). Primers and sequences are collects in Supplementary Table 2.

**Microinjection data**

CrispR/Cas9 Technology was applied to obtain the desired allele following the Yang Protocol (40). After this procedure we obtained 39 living pups. Of those pups, two mosaicisms (from a total of 8) were the desired allele, and one of them can transmit both the edited loxp sites to the progeny. To easily genotype founders and successive generations short range PCR was performed between both the inserted loxp sites (data not shown), led that every amplicon in the synthetic allele is 40 bp bigger in size, due to Loxp and restriction enzyme recognition sequence inserted (primers in Supplementary Table 2). Synthetic and wt amplicons bands were sequenced confirming the correct loxp integration events (data not shown). Cis loxp’s configuration of the conditional allele was then confirmed as described by Yang et al. (40): in brief Long Range PCR was performed outside the two inserted loxp sites, then the PshA1 restriction enzyme site inside the targeting allele was used to evaluate the cis configuration (Figure1B, C). Indeed, the expected bands profile in heterozygosity, homozigosity and in WT littermate (Figure 1B) is only obtained in “cis” configuration of the loxp’s sites. In addition, sequence was performed on the band obtained from the p21 floxed Homozygotes mouse (Figure 1 C line “Homo”), confirming the correct editing.

**BEHAVIOUR**

**COGNITIVE BEHAVIOUR**

**Open field.** At the beginning of each trial, each mouse was placed in the center of a circular white open field arena having a diameter of 100 cm, for 20 min. The arena was cleaned with 70% ethanol to avoid cue smell between each trial. The room was brightly lit, which in combination with the exposure to novel environment and open space induce anxiety in rodents. Thus, anxious behaviours were measured by time spent in center vs. time spent in the periphery of the open field arena. Locomotor activity was measured by distance travelled and average speed over the 20 min of exploration, where a large distance travelled and increased velocity are indicative of hyperactivity. Both thigmotaxis and locomotor activity were measured and analyzed using a specialized software with tracking system (Ethovision XT 8.5, Noldus Information Technology, USA). This test was performed 56 days after TAM in mice under physiological conditions and 30 days after TAM in mice subjected to TBI.

**Elevated Plus Maze.** The Elevated Plus Maze (EPM) consisted of a maze with four arms – two open arms without walls and two enclosed arms with walls 30 cm in height, 66 cm in length, and 10 cm in width. Each arm of the maze was attached with firm metallic legs (45 cm in height). Each mouse was placed in the central arm crossing area (10×10 cm) and was given 5 min to freely explore on the EPM. The maze was cleaned with 70% ethanol prior to the placement of each mouse. This test was performed 56 days after TAM in mice under physiological conditions.

**Morris water maze (MWM).** Mice were placed in a circular white pool (diameter 145 cm) filled with 23±2° C water made opaque by the addition of atoxic acrylic white color (Giotto, Italy). An escape platform (diameter 8 cm) with a rough surface was placed in the middle of the NW quadrant 20 cm from the side walls. It was submerged 0.5 cm under the water level. The pool located in a room uniformly lighted by four lamps (25 W each) was surrounded by several extra-maze cues. The water maze was surmounted by a video camera whose signal was relayed to a monitor and to the image analyzer (Etho VisionXT, Noldus, The Netherlands). The protocol consisted of a 16-trial Place phase and a 1-trial Probe phase. During the Place phase, mice from the two experimental groups were trained to locate a hidden platform using distal cues on four blocks of 90 s-trials each day for 4 days. During the 15-20 min inter-trial interval mice were put in their home cages. At the beginning of each trial, mice were gently released into the water from pseudo-randomly varied starting points and could swim around to find the hidden platform. Mice that did not locate the platform within 90 s were gently guided there by the experimenter. After mice climbed the platform, they were allowed to remain on it for 30 s. On the 5th day, spatial memory was then assessed during the Probe phase consisting of a trial performed 24 h after the final training trial. During the Probe phase the hidden platform was removed, and the mice were allowed to search for it for 45 s. To evaluate spatial learning and memory the following MWM parameters were analyzed: total distance swum (cm) and time spent (s) to reach the hidden platform, mean velocity (cm/s), and percentage of peripheral distance (i.e. distance traveled in a 20 cm-peripheral annulus) during the Place phase; percentage of distance swum and time spent in the previously rewarded quadrant during the Probe phase. This test was performed 56 days after TAM in mice under physiological conditions.

**IMMUNOHISTOCHEMISTRY**

Sections were initially washed with 0.1 M glycine for 10 min, followed by permeabilization using 0.3% Triton X-100 in PBS for another 10 min. The sections were then incubated for 30 min in a blocking solution that contained 3% normal donkey serum (NDS) in 0.3% Triton X-100 in PBS to saturate the specific sites, followed by incubation with the same blocking solution that contained primary antibodies for 16–18 h at 4 ◦C. The primary antibodies used were goat polyclonal antibodies, which were used against DCX (Santa Cruz Biotechnology, Dallas, TX, USA; Cat# Sc-8066; 1:300); SOX2 (Santa Cruz Biotechnology, Dallas, TX, USA; Cat# Sc-17320; 1:300) and NeuroD1 (R&D, Cat#AF2476, 1;300) a rabbit monoclonal antibodies were used against Ki67 (Lab Vision, South San Francisco, CA, USA, Cat# RM-9106-S; 1:150) and c-Fos (Millipore Cat# PC38, 1:200), whereas mouse monoclonal antibodies were used against GFAP (Sigma, St. Louis, MO, USA, Cat# G6171; 1:500) and NeuN (Millipore Cat# MAB377; 1:300). The detection of BrdU-positive cells consisted of denaturing DNA with 2N HCl for 45 min at 37 ◦C to facilitate antibody access. The sections were then incubated with 0.1 M sodium borate buffer at pH 8.5, followed by overnight incubation at 4 ◦C with a rat anti-BrdU primary antibody (Abcam, Cambridge, UK, Cat# ab6326; 1:300) diluted in TBS that contained 0.1% Triton, 0.1% Tween, and 3% normal donkey serum (blocking solution). To observe primary antibody binding, donkey secondary antibodies against rat (BrdU) and rabbit (Ki67) and goat (DCX) conjugated to Cy3 (Jackson ImmunoResearch, West Grove, PA, USA; 1:200 in PBS), against rabbit (c-Fos), goat (DCX, SOX2) and mouse (GFAP, NeuN) antibodies conjugated to Cy2 (Invitrogen, San Diego, CA, USA; 1:300 in PBS) and against rabbit (c-Fos), goat (DCX and SOX2) conjugated with Alexa-647 (Invitrogen, San Diego, CA, USA; 1:300 in PBS) were used. Nuclei were observed by incubating sections with Hoechst (1:500).

**CELL COUNTING**

Quantification of cells labelled with different markers in the DG has been obtained in every sixth coronal section (6 sections per brain) spanning the dorsal and ventral hippocampus. Pictures were acquired using confocal microscope (Leica SP5) and maximal intensity projection of three optical sections (10 μm thick in total) taken and quantified using ImageJ Software. Neurogenesis quantifications were carried out on one complete DG series per animal considering the subgranular zone or the whole thickness of the granular cell layer (immature and mature neurons) of both the dorsal and ventral hippocampus. The counting of DCX+ cells was carried out taking into consideration the different types of DCX-positive neuroblasts. We first counted the DCX+ cells that had a clearly recognizable nucleus that co-localized with the nuclei marker Hoechst. Furthermore, we counted both DCX+ cells containing vertical dendrites that extended towards the molecular layer, and those with short dendritic processes that extended parallel to the sub-granular zone.

***IN VITRO* ANALYSIS**

To perform the neurosphere assay, cells isolated from DG were isolated by enzymatic digestion (1.33 mg/ml trypsin, 0.7 mg/ml hyaluronidase, and 0.2 mg/ml kynurenic acid) for 30 min at 37°C pipetting every 15 min with a small-bore Pasteur pipette allowing mechanical dissociation. In every tube, 5 ml of DMEM/F12 were added to consent tissues precipitation: after 10 min at room temperature, the superior phase was removed, leaving a small volume on the tip of the tube. Then, samples were centrifuged at 200 g for 5 min, supernatant was removed, and the pellet was pipetted 40–50 times to fully dissociate cells. Neurospheres were grown in a humidified incubator at 37°C in 5% CO2 and cultured in DMEM/F12 medium supplemented with B27 and EGF (20 ng/ml) and bFGF (10 ng/ml). Cells were plated at 10 cells/µl in 24-well (0.5 ml/well) uncoated plates in growth medium. The total number of neurospheres was counted after 7 days in vitro (7 DIV).

**EXPRESSION ANALYSIS BY qRT-PCR**

After RNA extraction, it was was quantified with NanoDrop (Thermo Scientific NanoDrop 2000C). For mRNA analysis, total RNA (500 ng) was retro-transcribed with a retrotranscription kit (Thermo Fisher Scientific Cat#: 8080234) by using random hexamers. Quantitative Real Time PCR (qRT-PCR) analysis was performed with SYBR Green Master Mix (PowerUp - Applied Biosystems) and p21 primer pairs designed with Primer3 Input software (primer3.ut.ee). The murine expression primer used in this study spanned an exon-exon junction. The reactions were run on 7900HT ABI prism PCR machine (Applied Biosystems).
