# Supplementary material for: Essential role of p21Waf1/Cip1 in the modulation of post-traumatic hippocampal Neural Stem Cells response
Source: Stem Cell Res Ther. 2024 Jul 6;15:197. doi: 10.1186/s13287-024-03787-0 (PMC11227726; doi:10.1186/s13287-024-03787-0)
Supplement: Supplementary file 2 — Additional file 2. Supplementary Figures and Tables. [file 13287_2024_3787_MOESM2_ESM.docx]

**ADDITIONAL FILES 2**

**
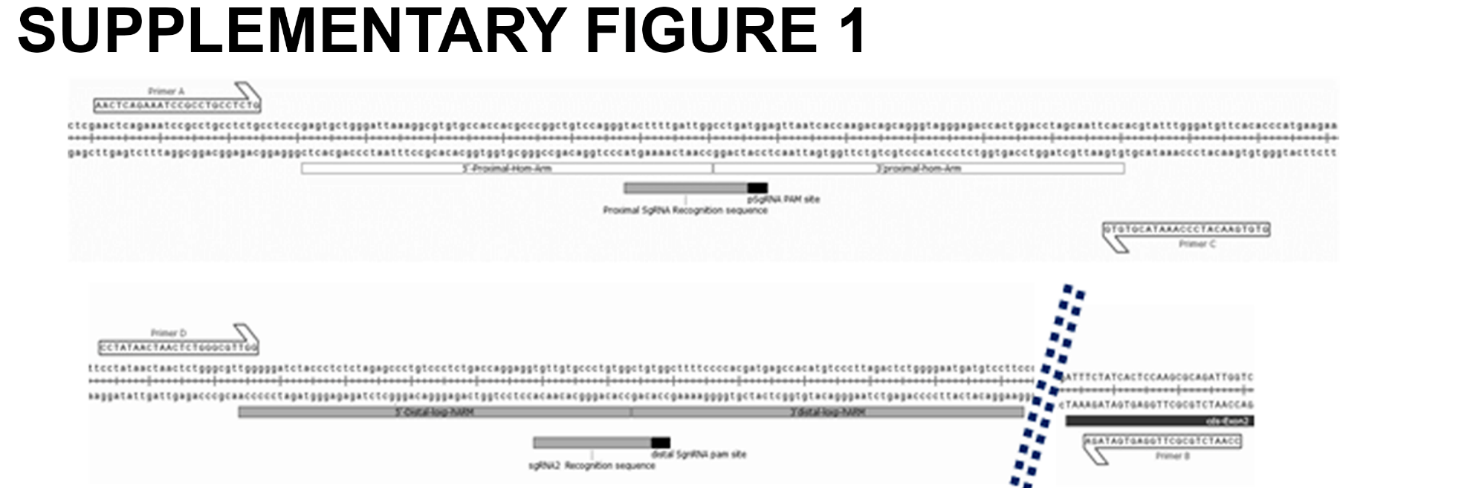
**

**SUPPLEMENTARY FIGURE 1.** **Design of the two CrispR/Cas9 target loci.** SgRNA’s are shown with the pam sites in black. PCR for loxps genotyping was performed: Proximal Loxp: Primers A+C: amplicon 171 bp in WT; 211 bp synthetic. Distal Loxp: Primers D+B: amplicon 207 bp in WT: 247 synthetic.


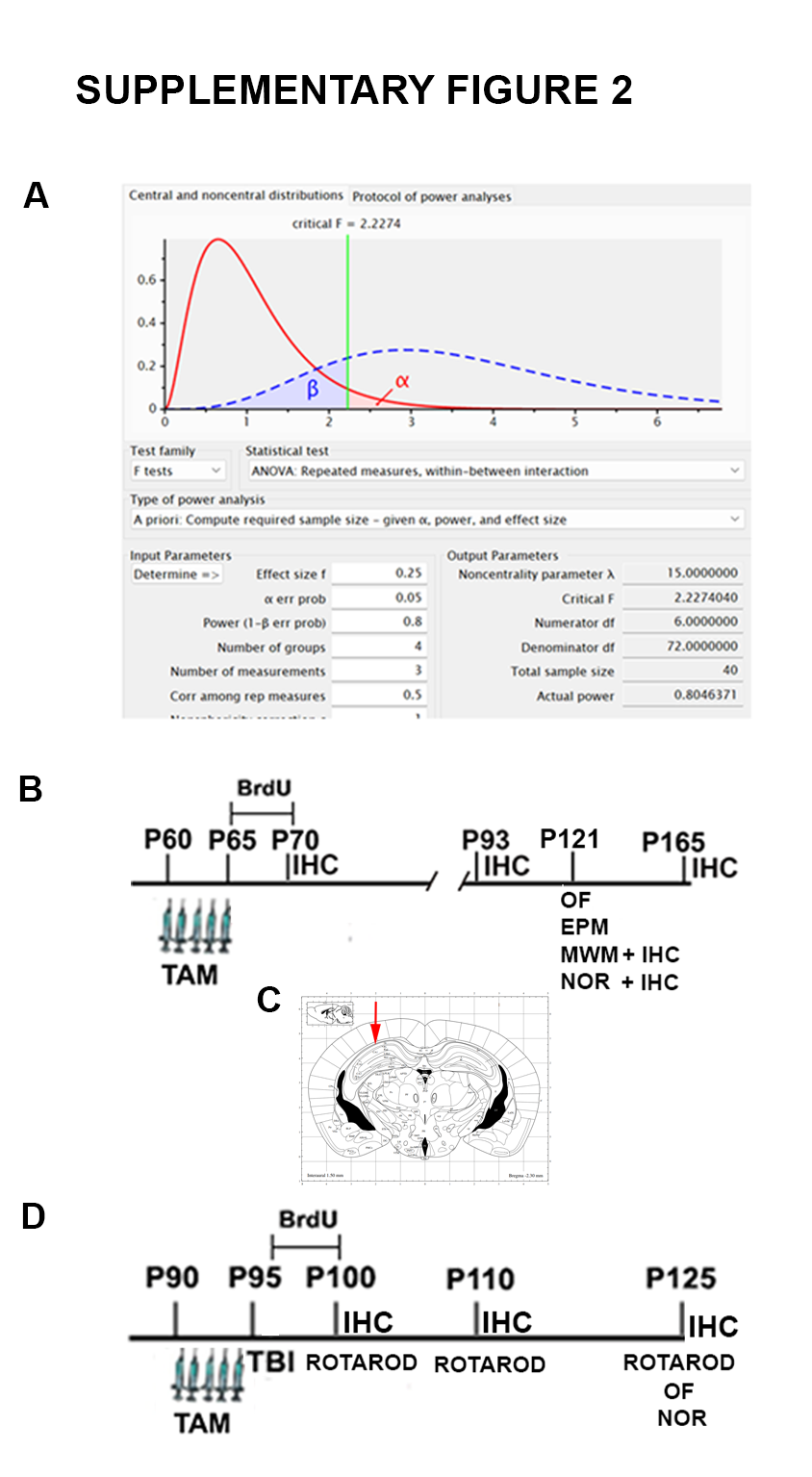


**SUPPLEMENTARY FIGURE 2. (A)** Power Analysis carried out to determine the number of animals needed for behavioral studies. **(B)** Scheme representing experiments carried out on animals not subjected to CCI/SHAM operations. **(C)** Scheme illustrating the coordinates of the brain region injured by CCI. The red arrow indicates the approximate point of impact within the brain. (D) Scheme representing experiments carried out on animals subjected to CCI/SHAM operations.


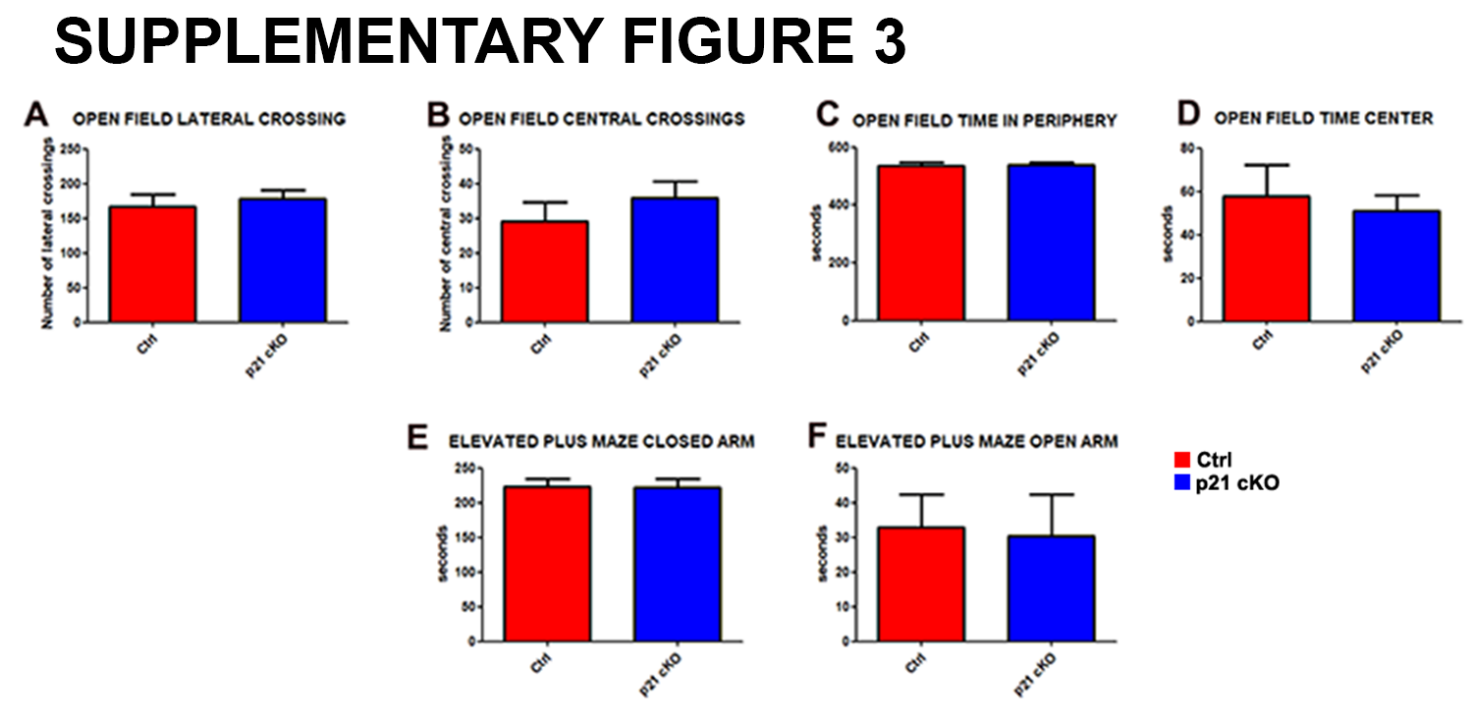


**SUPPLEMENTARY FIGURE 3. Effect of p21 deletion in the Open Field and Elevated Plus Maze (A-D).** Graphs representing that the number of crossings (A-B) and the time spent in the different regions of arena of Open Field (C-D) are similar in the Ctrl and p21 cKO mice (p21 cKO vs Ctrl: Mann Whitney Test, p> 0.05). **(E-F)** Histograms show that the Ctrl and p21 cKO mice spent comparable time in the closed (E) and opened (F) arms of the Elevated Plus Maze (p21 cKO vs Ctrl: Mann Whitney Test, p> 0.05 for both parameters).


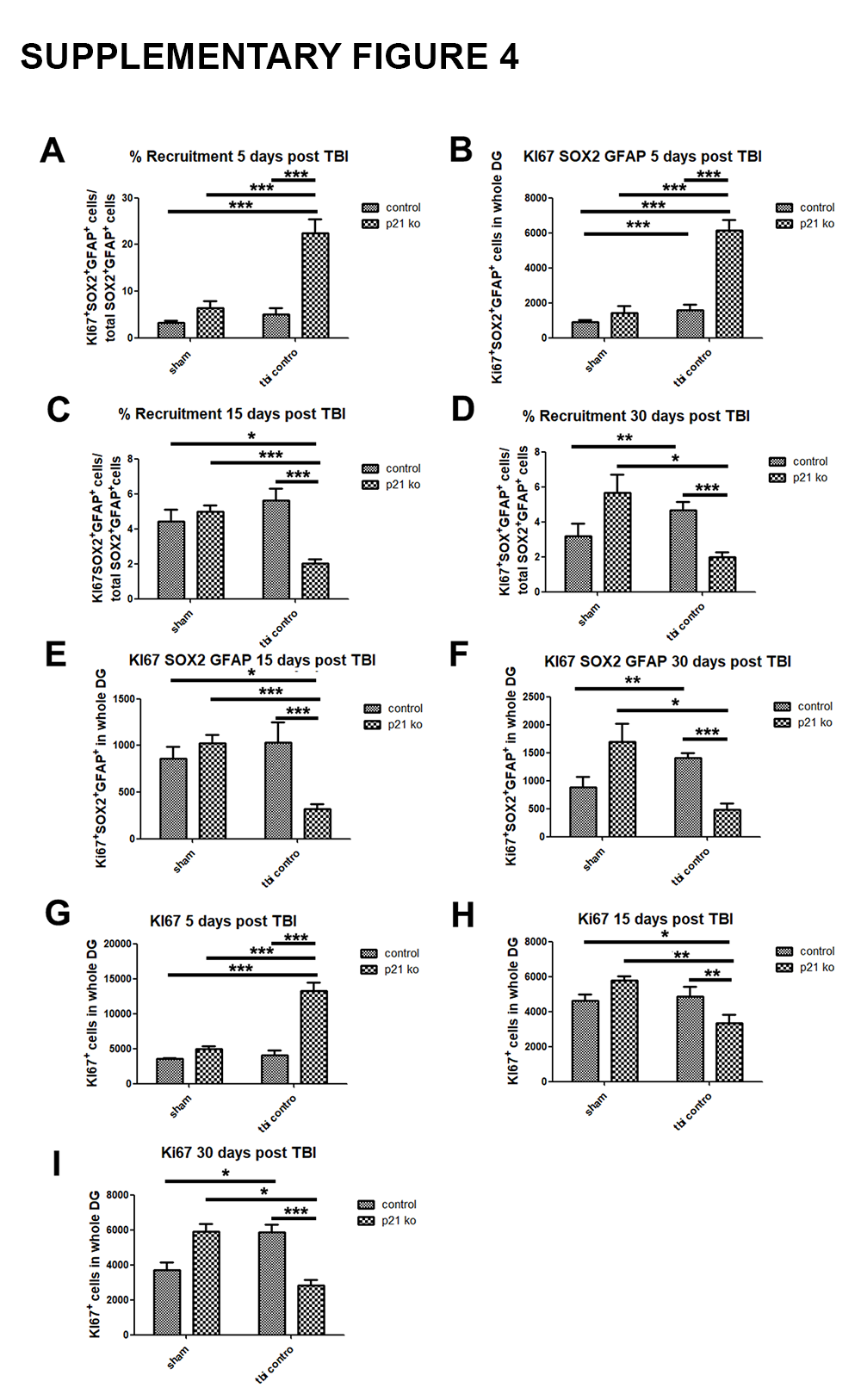


**SUPPLEMENTARY FIGURE 4.** (**A-B**). **Effect of deletion of p21 in contro-lateral NSCs during post-traumatic neurogenic response** 5-days post TBI, the two-way ANOVA indicates an impressive increase in the recruitment rate (**A**) in the contralateral DG of p21 cKO mice, with respect to the other 3 experimental conditions (genotype x TBI interaction: F_(1,29)_ = 23,2, p < 0.001, followed by Bonferroni post-hoc, cKO TBI vs Ctrl SHAM, cKO SHAM and Ctrl TBI p < 0.001) and of NSCs proliferation (**B**, contro-lateral, genotype x TBI interaction: F_(1,29)_ = 39,78 p < 0.001, followed by Bonferroni post-hoc, cKO TBI vs Ctrl SHAM, cKO SHAM and Ctrl TBI p < 0.001). Notably, we even observed a rise of NSCs activation in TBI vs SHAM control mice (contro-lateral: Ctrl TBI vs Ctrl SHAM p <0,001). On the contrary we observed a drastic reduction of NSC recruitment in the contralateral DG of p21 cKO both 15 days (**C**, genotype x TBI interaction: F_(1,35)_ = 8,9 p < 0.01, followed by Bonferroni post-test, cKO TBI vs cKO SHAM and Ctrl TBI p < 0.001, vs Ctrl SHAM p < 0.05) and 30-days post TBI (**D**, genotype x TBI interaction: F_(1,32)_ = 10,6 p < 0.01, followed by Bonferroni post-test, cKO TBI vs cKO SHAM p > 0.05, vs Ctrl TBI p < 0.001). Similarly, we observed a reduction of NSCs proliferation at both timepoints (**E**, 15 day post-TBI: genotype x TBI interaction: F_(1,37_) = 8,3, p < 0.01, followed by Bonferroni post-test, cKO TBI vs cKO SHAM and Ctrl TBI p < 0.001, vs Ctrl SHAM p < 0.05; **F**, 30 days post-TBI: genotype x TBI interaction: F_(1,32)_ = 14,7 p < 0.001, followed by Bonferroni post-test, cKO TBI vs cKO SHAM p > 0.01, vs Ctrl TBI p < 0.001). Moreover, we observed a strong increase of Ki67+ cells in the DG of p21 cKO 5 days following the TBi (**G**, genotype x TBI interaction: F_(1,75)_ = 35,9 p < 0.001, followed by Bonferroni post-test, cKO TBI vs Ctrl SHAM, cKO SHAM and Ctrl TBI p < 0.001) and a strong Ki67^+^ reduction 15 days (**H**, genotype x TBI interaction: F_(1,85)_ = 11,9 p < 0.001, followed by Bonferroni post-test, cKO TBI vs Ctrl SHAM p < 0.05, vs cKO SHAM p < 0.001 and vs Ctrl TBI p < 0.01) and 30 days after TBI (**I**, genotype x TBI interaction: F_(1,30)_ = 40,5 p < 0.001, followed by Bonferroni post-test, cKO TBI vs cKO SHAM p > 0.05, vs Ctrl TBI p < 0. 01). Statistical significance: *p < 0.05, **p < 0.01, ***p < 0.001. Two-way ANOVA analysis, by Bonferroni post hoc tests.

**
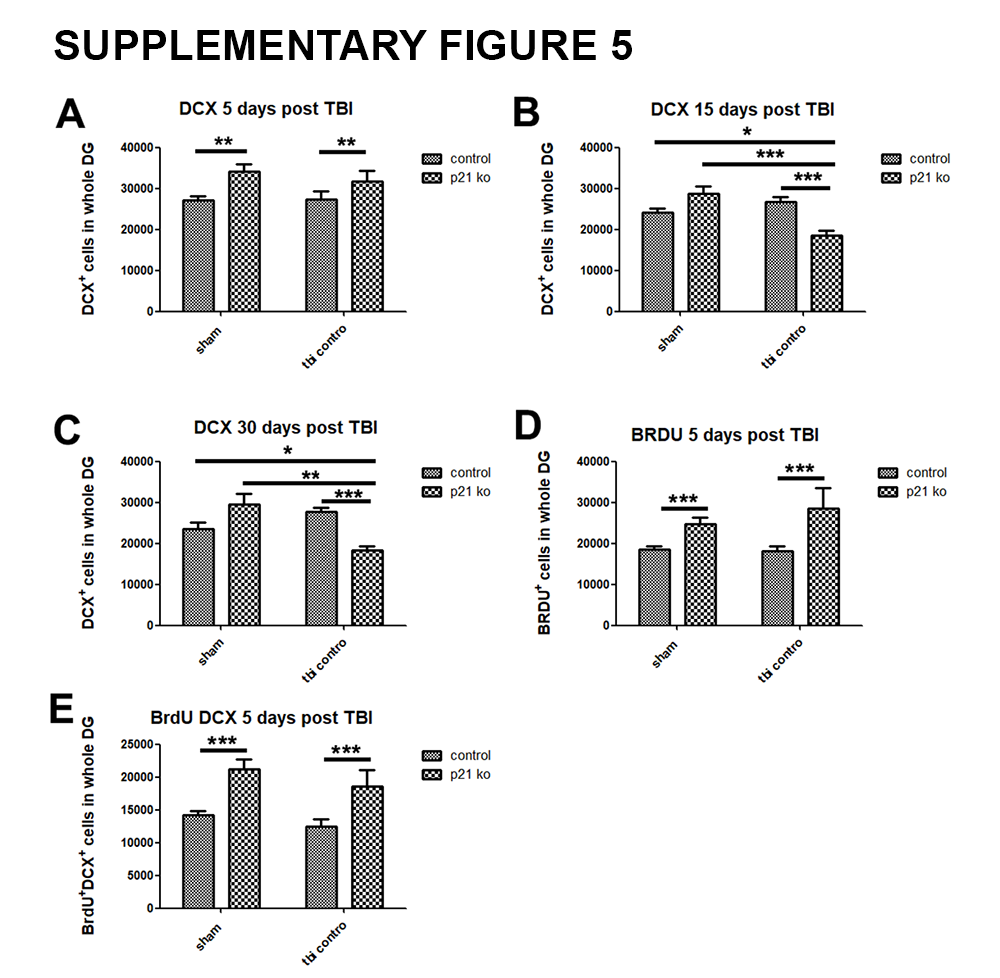
**

**SUPPLEMENTARY FIGURE 5. Effect of deletion of p21 in contro-lateral neurogenesis during post-traumatic neurogenic response** 5 days post-TBI, we observed an increase of DCX neuroblast in the contralateral DG in p21 cKO mice (genotype effect F_(1,70)_ = 9,9, p < 0.01) but a reduction of DCX+ cells both 15 days (**B,** genotype x TBI interaction: F_(1,47)_ = 9,7 p < 0.01, followed by Bonferroni post-test, cKO TBI vs Ctrl SHAM p < 0.001, vs cKO SHAM p < 0.05 and vs Ctrl TBI p < 0.001, Ctrl TBI vs Ctrl SHAM p < 0.001) and 30 days after TBI (**C**, genotype x TBI interaction: F_(1,29)_ = 14,9 p < 0.001, followed by Bonferroni post-test, cKO TBI vs Ctrl SHAM p < 0.05, cKO SHAM p > 0.01, vs Ctrl TBI p < 0. 001; Ctrl TBI vs Ctrl SHAM p < 0.05). (**D**) 5 days post-TBI BrdU- expressiong cells in the controlateral DG are significantly increased in p21 cKO compared to control mice (genotype effect: F_(1,36)_ = 16,53, p < 0.001) as well as newborn neuroblasts (**E**, genotype effect: F_(1,35)_ = 24, p < 0.001). Two-way ANOVA analysis, by Bonferroni post hoc tests.

**
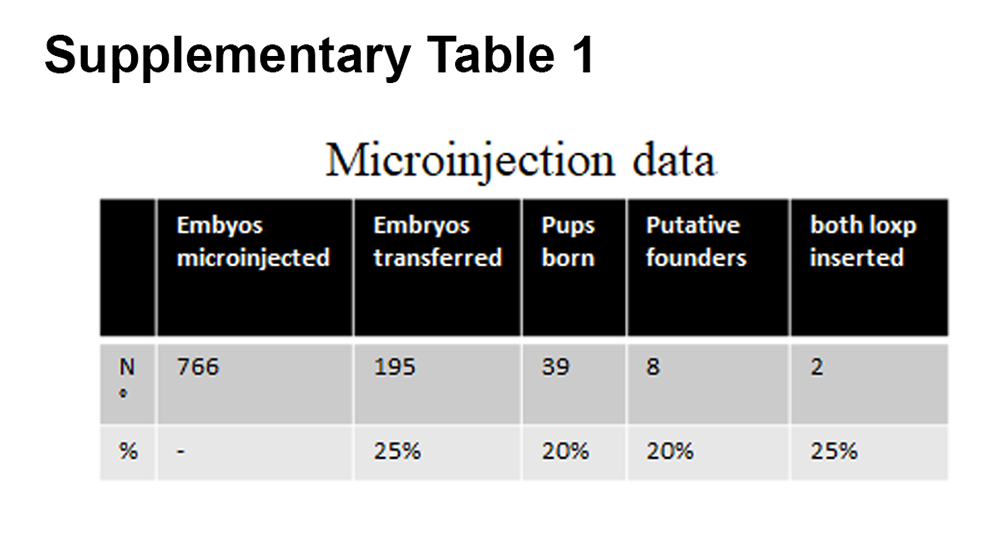
**

**SUPPLEMENTARY TABLE 1.** Microinjection data, retrieved from three session experiments.


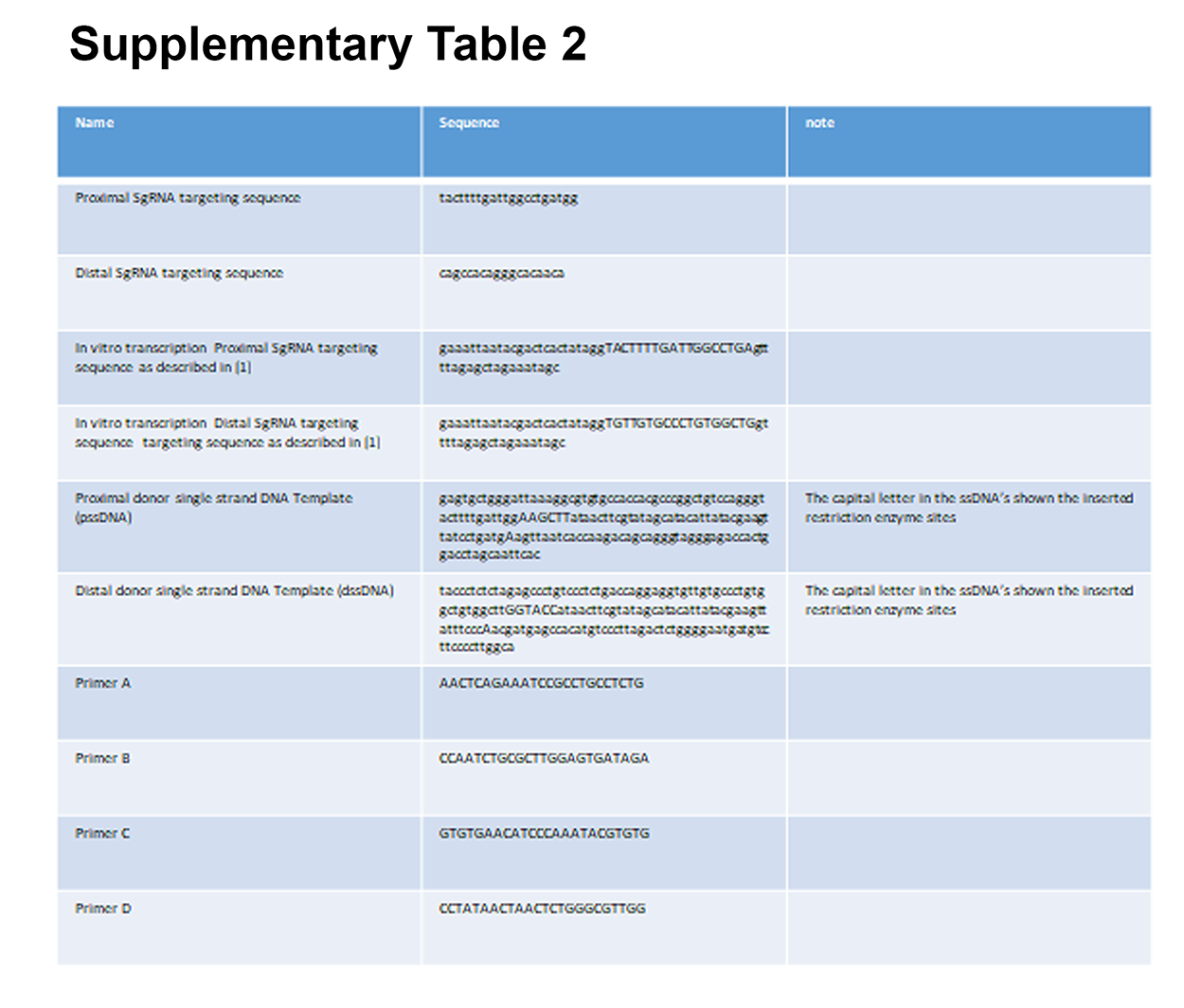


**SUPPLEMENTARY TABLE 2.** Primers and sequences used for the p21 cKO mouse model generation.

**Supplementary Table 3**

**List of murine expression primers used in this study**

| **Gene Symbol** | **Forward** | **Reverse** |
| --- | --- | --- |
| p21 | TGAGCGGCCTGAAGATTCCC | GATAGAAATCTGTCAGGCTGGTCTGC |
| Ccnd1 | TCCTCTCCAAAATGCCAGAG | GGGTGGGTTGGAAATGAAC |
| Ccnd2 | TTCAGCAGGATGATGAAGTGA | AGCAGAGCTTCGATTTGCTC |
